# Supplementary material for: The archetypal gene transfer agent RcGTA is regulated via direct interaction with the enigmatic RNA polymerase omega subunit
Source: Cell Rep. 2022 Aug 9;40(6):111183. doi: 10.1016/j.celrep.2022.111183 (PMC9638019; doi:10.1016/j.celrep.2022.111183)
Supplement: Document S1. Figures S1–S7 [file mmc1.pdf]

Cell Reports, Volume *40*

Supplemental information

The archetypal gene transfer agent RcGTA is regulated via direct interaction with the enigmatic RNA polymerase omega subunit

David Sherlock and Paul C.M. Fogg

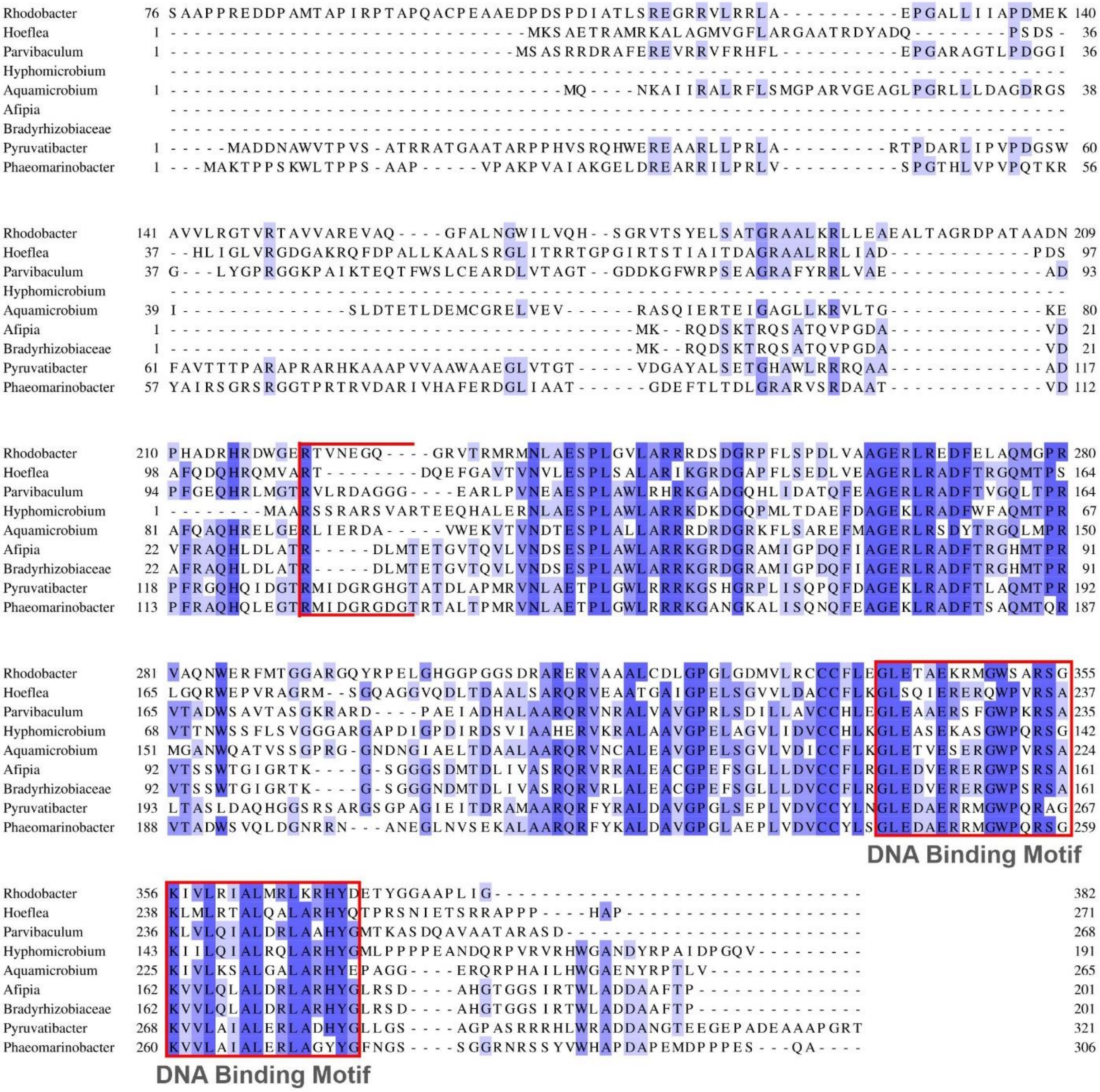

Figure S1. Alignment of the *R. capsulatus* GafA C-terminal extended domain with Hyphomicrobiales counterparts. Related to Figure 3. The top four hits against fully assembled Hyphomicrobiales genomes were chosen from separate BLASTp and PSI-BLAST sequence similarity searches with an *R. capsulatus* GafA query. Conservation is indicated with the Jalview percentage identity colour scheme. The predicted C-terminal DNA binding domain is boxed and annotated to highlight increased sequence conservation. The open box indicated the beginning of the C-terminal concise constructs.

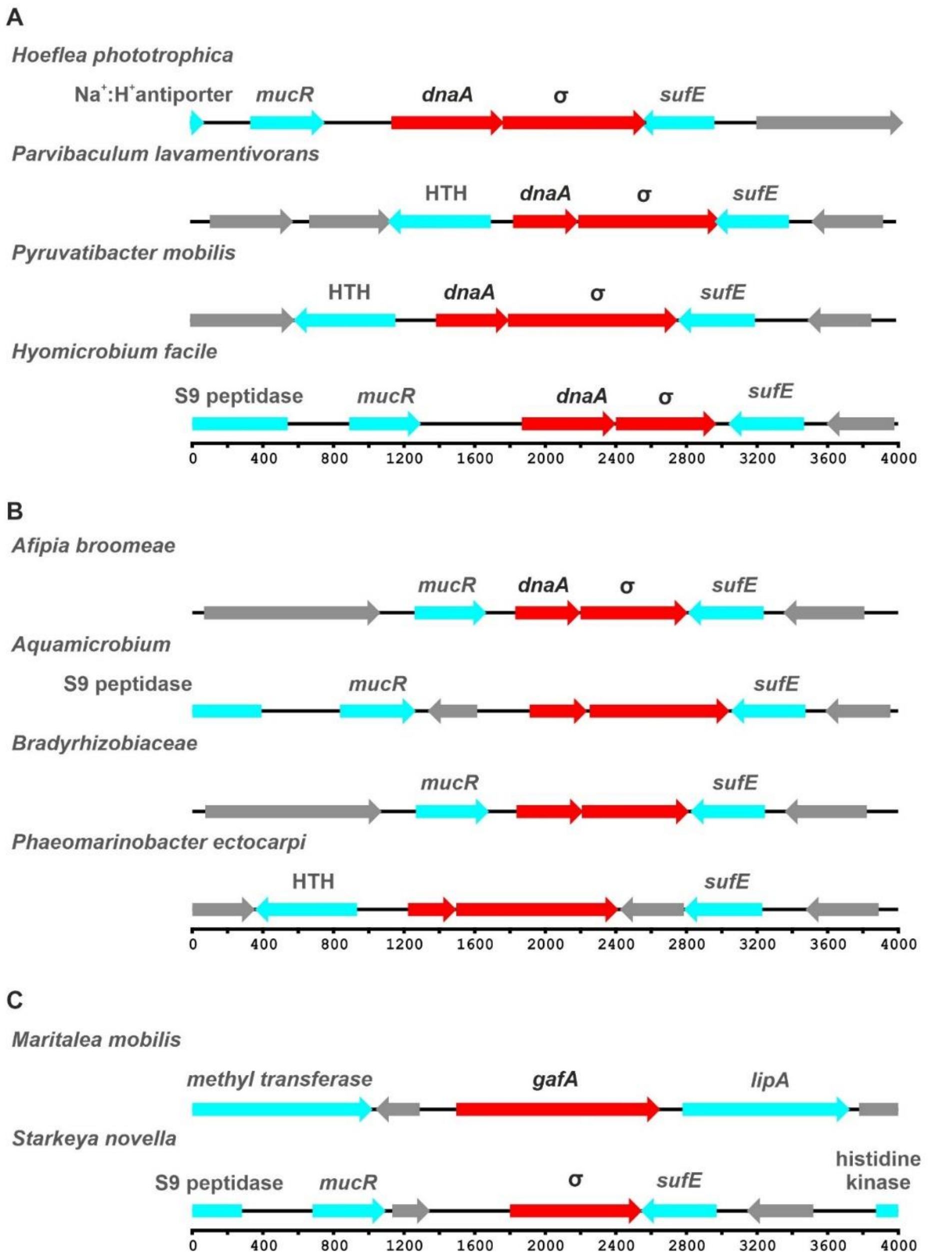

Figure S2. Synteny plots for Hyphomicrobiales GafA homologues. Related to Figure 3. The top four **A.** BLASTp and **B.** PSI-BLAST hits for *R. capsulatus* GafA against fully assembled Hyphomicrobiales genomes. Sequence matches mainly occurred for the GafA C-terminal region only with genes annotated as DUF6456 domain-containing proteins. The matched Hyphomicrobiales genes are annotated here using the HHPRED prediction of a Sigma factor-like domain (σ) and the ORF is coloured red. The upstream dnaA-like ORF is also coloured red. Flanking genes with predicted function are cyan, hypothetical proteins of unknown function are grey. **C.** Two exceptions are shown where either a full-length match was obtained but with Rhodoabcterales-like synteny (*Maritalea*) or the *dnaA* gene was absent with otherwise Hyphomicrobiale-like synteny (*Starkeya*). Scale bars are provided below each panel in bases.

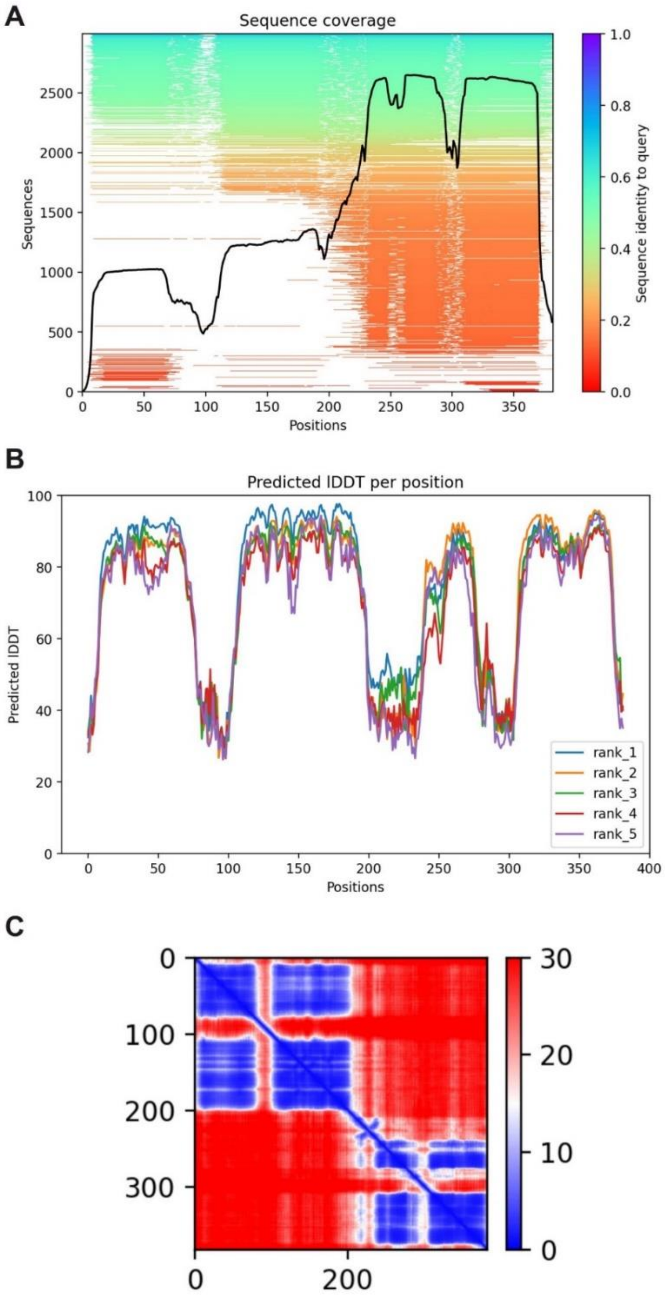

Figure S3. Confidence outputs for *R. capsulatus* GafA structure prediction. Related to Figure 3. A. The jackhmmer method was used on the Alphafold server to align GafA to related proteins and the multiple sequence alignment coverage plot is shown. Aligned sequence coverage is depicted as a line chart and sequence identity is colour coded as shown in the legend. **B.** AlphaFold output plot showing the predicted local Distance Difference Test score (pIDDT) confidence metric. Amino acid positions are shown on the X-axis. **C.** Predicted Aligned Error for each amino acid position labelled on the X and Y-axes. Error is shown on a scale of 0-30, and colour coded as shown in the legend. Clear drop-offs in model confidence can be seen between predicted domains, but each domain is has strong scores typically >80.

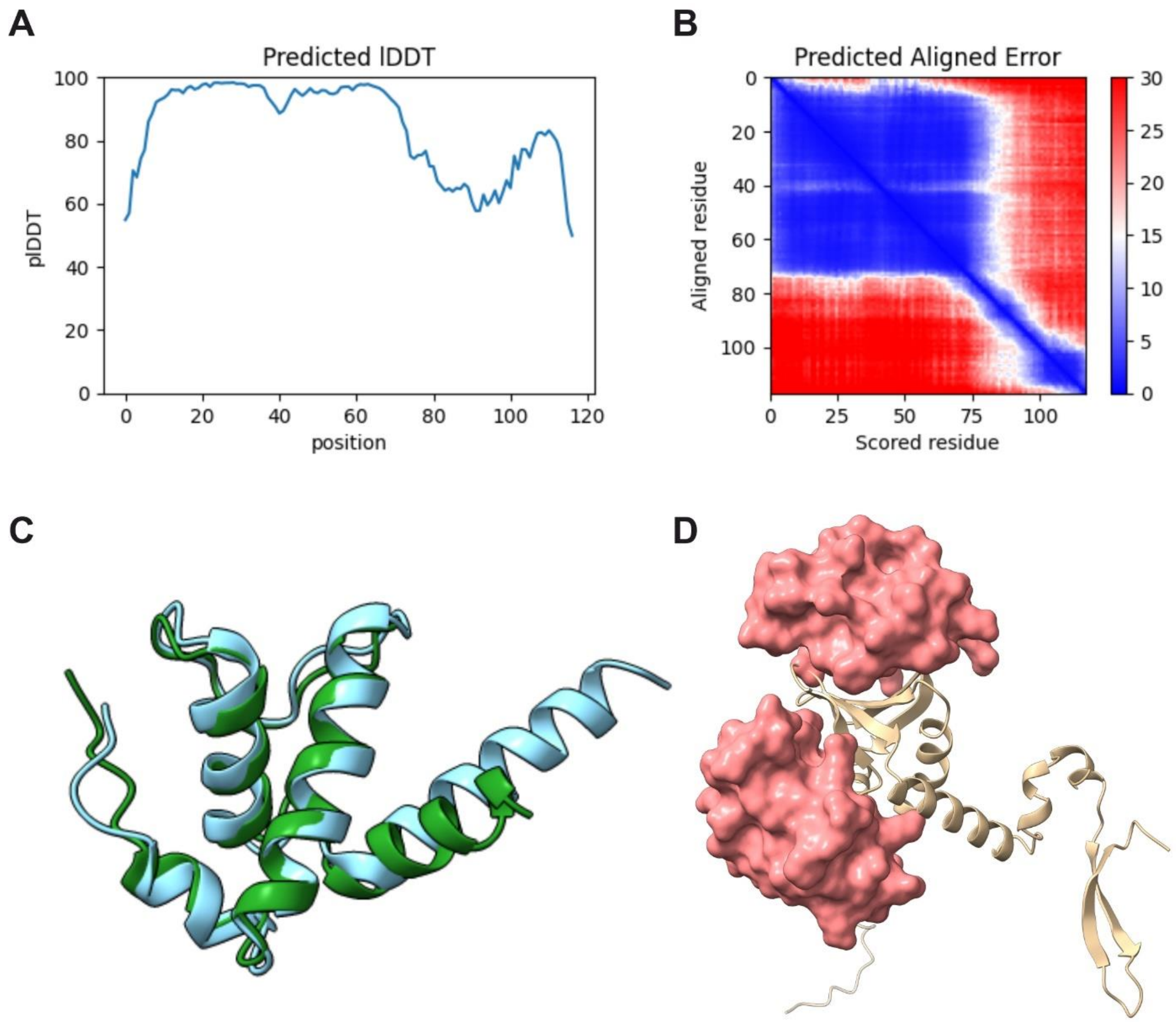

Figure S4. Predicted structure of *R. capsulatus* Rpo-ω protein and its interaction with GafA. Related to Figure 3. A & B. AlphaFold output plots showing the predicted local Distance Difference Test score (pIDDT) confidence metric and Predicted Aligned Error for each amino acid position. A clear drop-off in model confidence, domain packing and broader topology is observed from approximately residue 70 onwards. **C.** AlphaFold predicted *R. capsulatus* Rpo-ω structure trimmed to residues 1-71 (green) and overlaid with *E. coli* Rpo-ω, PDB: 6ALF (pale blue). **D.** LZerD protein docking predictions for GafA-CenN and Rpo-$\omega^{1\text{-}71}$. The two Rpo-ω surface structures shown are representatives of the two centroid clusters that comprise the top ten interaction models. The upper location in contact with the β-sheet was favoured by 6 out of 10 models including the top ranked (rank sum = 47).

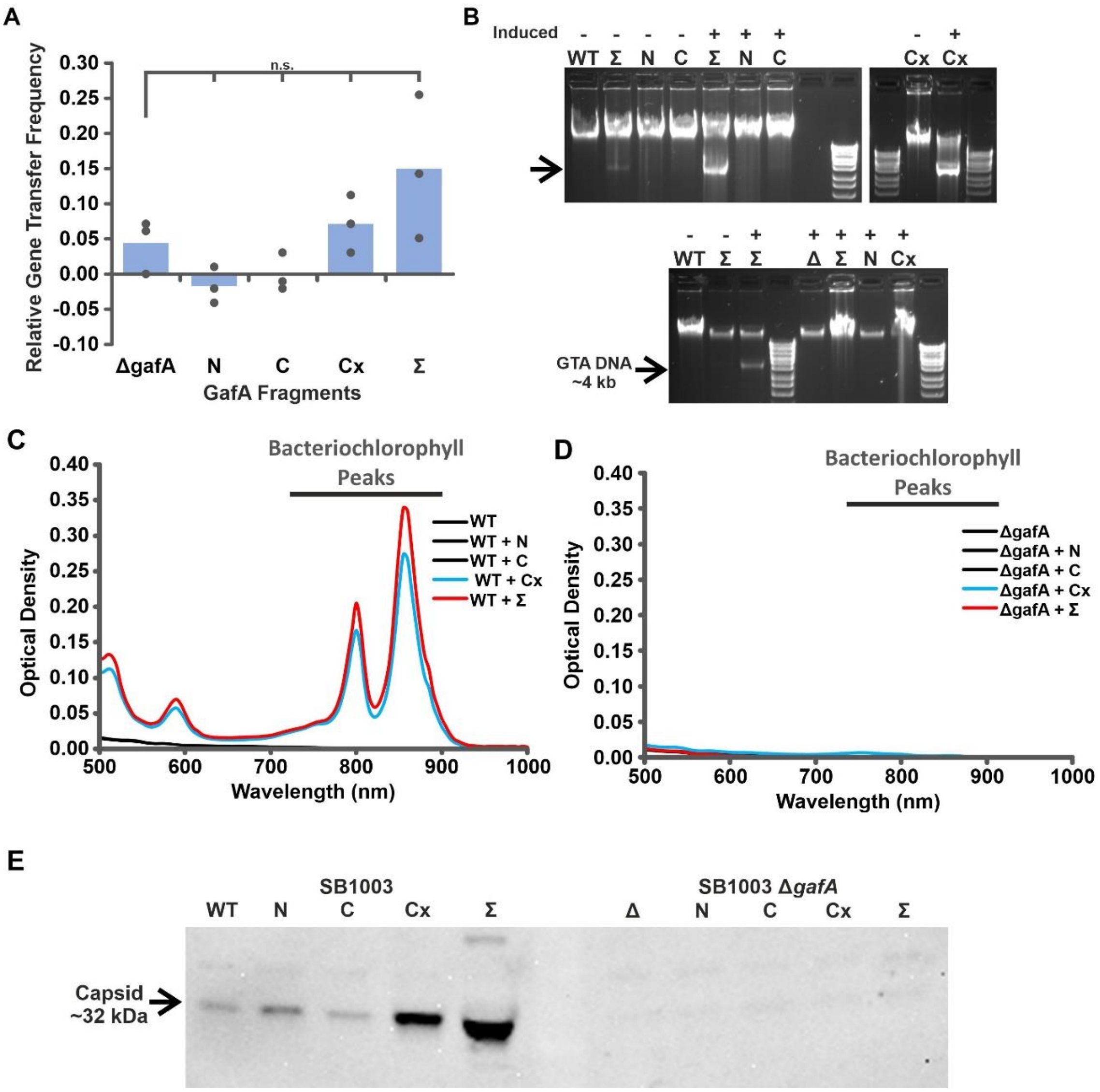

Figure S5. RcGTA production phenotypes after *in trans* expression of GafA full length and truncated proteins. Related to Figure 4. In all panels, SB1003 wild-type and a Δ*gafA* derivative were complemented with empty pQF vector (WT or Δ) or pQF containing truncated *gafA* genes as indicated (*gafA*-N, *gafA*-C, *gafA*-Cx, *gafA*-Σ). **A.** Chart of the frequency of rifampicin gene transfer from *R. capsulatus* SB1003 Δ*gafA* donor strains complemented *in trans* with the indicated pQF vectors, N = 3. **B.** Total intracellular DNA content showing the presence or absence of characteristic 4 kb RcGTA DNA. **C.** Mean absorbance trace of *R. capsulatus* SB1003 supernatant or **D.** SB1003 Δ*gafA* supernatants in the 500-1000 nm wavelength range. Complementation *in trans* the pQF plasmid containing full-length *gafA* is represented by a red line, with *gafA-Cx* is represented by a cyan line and all other constructs (pQF-empty, *gafA*-N and *gafA*-C) are shown in black. N=6 except Δ*gafA* + Cx N=4. Distinctive bacteriochlorophyll peaks indicating cells lysis are annotated. **E.** Representative western blot of concentrated supernatant from the indicated *R. capsulatus* strains using an α-RcGTA capsid antibody. See also Data S1.

A

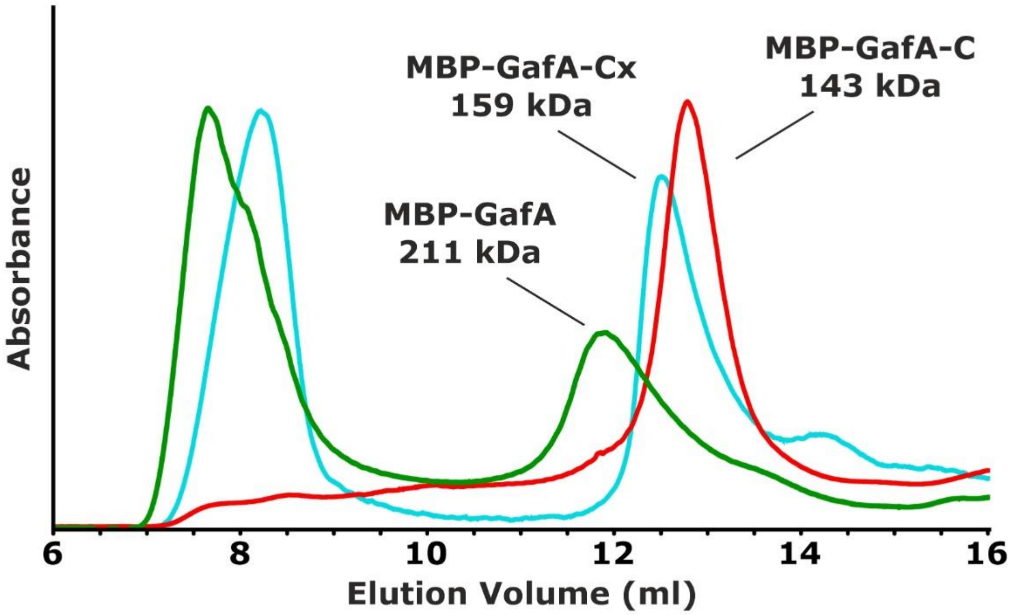

B

Protein	Elution Peak (ml)	Estimated MW (kDa)	Monomer Size (kDa)	Ratio
**MBP-GafA-Cx**	12.54	158,779	75,806	2.1
**MBP-GafA-C**	12.78	142,537	61,119	2.3
**MBP-GafA**	11.91	210,774	85,159	2.5

Figure S6. Analytical gel filtration of GafA proteins. Related to Figure 6. A. Representative traces showing absorbance of GafA (green), GafA-Cx (cyan) and GafA-C (red) at 280 nm versus elution time from the column. Absorbance values are omitted on the Y-axis because the traces are scaled differently to improve comparability. **B.** Summary table of values plotted in part A, the estimated MW of the protein peaks, the calculated MW of each monomer and the ratio of observed MW to that of the monomer.

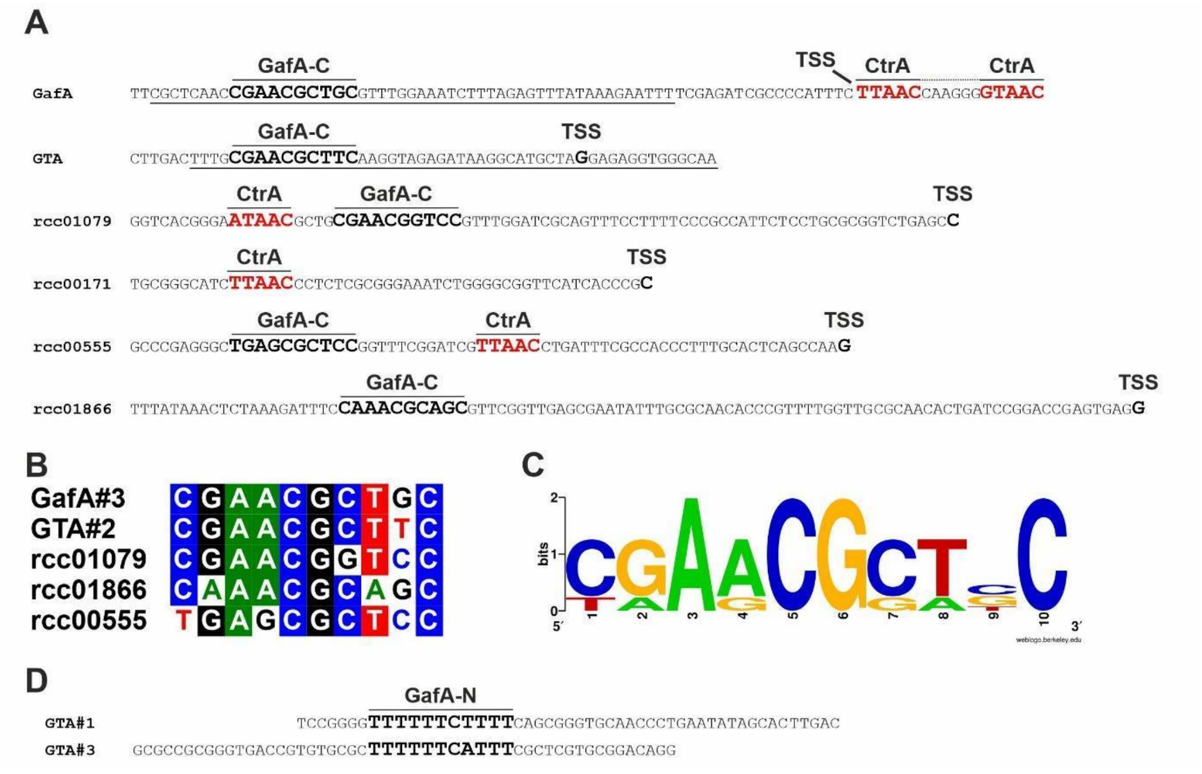

Figure S7. Predicted binding sites for GafA N/C-terminal DNA binding domains. Related to Figure 6. **A.** Schematic of RcGTA related promoters. Transcription start sites (TSS) were estimated based on published RNAseq data. Predicted CtrA binding sites/half-sites are highlighted in bold red and annotated, predicted GafA C-terminal (GafA-C) DNA binding sites are highlighted in bold black and annotated. Underlined sequence indicates the region used for EMSA band shift assays. The five predicted GafA-C binding sites are depicted in **B.** an alignment and **C.** a Logo plot. **D.** The two oligo sequences that were specifically bound by the GafA N-terminal DNA binding domain (GafA-N) are shown with the putative binding site aligned, emboldened and annotated.
