# Supplementary material for: The archetypal gene transfer agent RcGTA is regulated via direct interaction with the enigmatic RNA polymerase omega subunit
Source: Cell Rep. 2022 Aug 9;40(6):111183. doi: 10.1016/j.celrep.2022.111183 (PMC9638019; doi:10.1016/j.celrep.2022.111183)
Supplement: Document S2. Article plus supplemental information [file mmc7.pdf]

Cell Reports

Article

The archetypal gene transfer agent RcGTA is regulated via direct interaction with the enigmatic RNA polymerase omega subunit

Graphical abstract

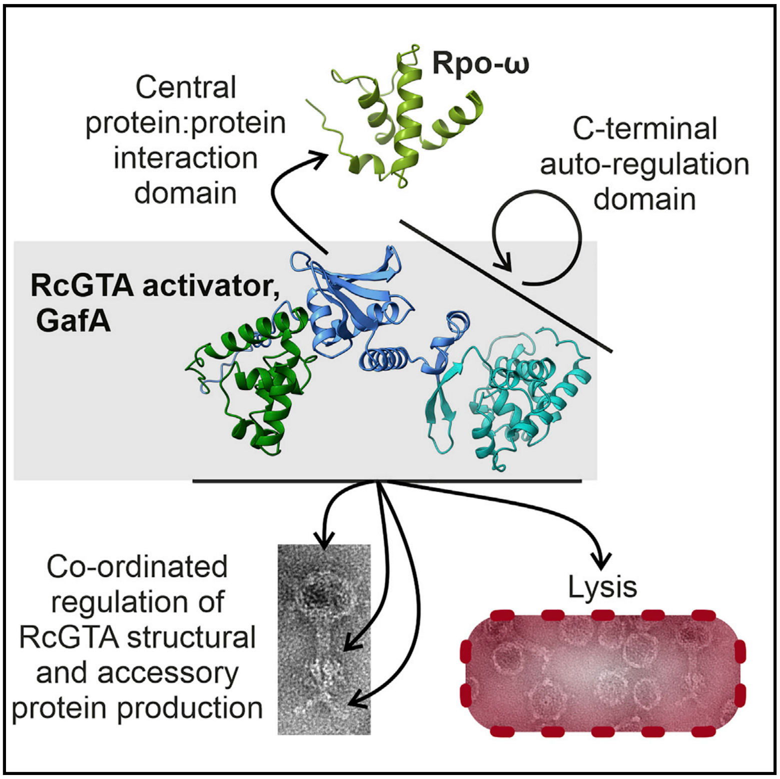

Highlights

- *Rhodobacter capsulatus* transcriptional factor GafA is a multi-domain protein
- Distal DNA-binding domains facilitate autoregulation and expression of RcGTA genes
- GafA controls RNAP promoter selection via interaction with the omega subunit
- Comparable *gafA* genes are present in the Hyphomicrobiales but split into two genes

Authors

David Sherlock, Paul C.M. Fogg

Correspondence

paul.fogg@york.ac.uk

In brief

Sherlock and Fogg show that the *Rhodobacter capsulatus* transcriptional factor GafA is a multi-domain protein. The C terminus binds to its own promoter for autoregulation, the central region directly interacts with RNA polymerase via the omega subunit, and the whole protein is required for activation of the virus-like gene transfer agent.

Sherlock & Fogg, 2022, Cell Reports *40*, 111183
August 9, 2022 © 2022 The Author(s).
https://doi.org/10.1016/j.celrep.2022.111183

CellPress

Cell Reports

CellPress
OPEN ACCESS

Article

The archetypal gene transfer agent RcGTA is regulated via direct interaction with the enigmatic RNA polymerase omega subunit

David Sherlock[1] and Paul C.M. Fogg[1,2,3,*]
[1]Biology Department, University of York, York YO10 5DD, UK
[2]York Biomedical Research Institute (YBRI), University of York, York YO10 5NG, UK
[3]Lead contact
*Correspondence: paul.fogg@york.ac.uk
https://doi.org/10.1016/j.celrep.2022.111183

SUMMARY

Gene transfer agents (GTAs) are small virus-like particles that indiscriminately package and transfer any DNA present in their host cell, with clear implications for bacterial evolution. The first transcriptional regulator that directly controls GTA expression, GafA, was recently discovered, but its mechanism of action has remained elusive. Here, we demonstrate that GafA controls GTA gene expression via direct interaction with the RNA polymerase omega subunit (Rpo-ω) and also positively autoregulates its own expression by an Rpo-ω-independent mechanism. We show that GafA is a modular protein with distinct DNA and protein binding domains. The functional domains we observe in *Rhodobacter* GafA also correspond to two-gene operons in Hyphomicrobiales pathogens. These data allow us to produce the most complete regulatory model for a GTA and point toward an atypical mechanism for RNA polymerase recruitment and specific transcriptional activation in the Alphaproteobacteria.

INTRODUCTION

Horizontal gene transfer by viruses and other mobile genetic elements is the major driver of rapid bacterial adaptation and spread of traits such as antibiotic resistance. Gene transfer agents (GTAs) are virus-like genetic elements that are similar to viruses, but instead of prioritizing the spread of their own genes, they package and disseminate any DNA within the host cell (Hynes et al., 2016; Lang et al., 2012; Shakya et al., 2017; Sherlock et al., 2019; Tamarit et al., 2018). Although GTAs usually package and transfer "random" fragments of DNA from their host to compatible recipients in headful fragments (Berglund et al., 2009; Esterman et al., 2021; Freese et al., 2017; Hynes et al., 2012; Sherlock et al., 2019), some species do exhibit bias toward certain regions of the genome (Berglund et al., 2009; Tomasch et al., 2018). Significantly, GTAs have been implicated in high-frequency spread of genes between bacteria (McDaniel et al., 2010), and an extensive survey of the function of thousands of bacterial genes indicated that GTA genes convey significant fitness benefits in multiple species under stress conditions (Kogay et al., 2019, 2020; Price et al., 2018).

The true prevalence of GTAs is not currently known, but a recent study identified homologs of the model *Rhodobacter capsulatus* GTA (RcGTA) is present in at least 50% of sequenced Alphaproteobacteria genomes, many of which had been misannotated as remnant prophages (Kogay et al., 2019, 2020; Shakya et al., 2017). The GTA genes are often dispersed at multiple genomic locations (Hynes et al., 2016; Motro et al., 2009), and coordinated expression initiates from a small subset of the bacterial population (Fogg, 2019; Fogg et al., 2012; Hynes et al., 2012; Québatte and Dehio, 2019). The timing and regulation of GTA production are tightly controlled by interlinked host regulatory circuits, including quorum sensing (Koppenhöfer et al., 2019; Leung et al., 2012), stringent response (Québatte et al., 2017; Westbye et al., 2017), SOS response (Kuchinski et al., 2016), cyclic diguanylate (c-di-GMP) (Pallegar et al., 2020a; 2020b), and the pleiotropic transcription factor CtrA (Lang and Beatty, 2000; Westbye et al., 2018). In *R. capsulatus*, these complex pathways are integrated via a specific GTA transcriptional regulator, GafA (Fogg, 2019), and a repeats in toxin superfamily-domain (RTX) extracellular repressor, *rcc00280* (Ding et al., 2019; Westbye et al., 2018). However, the precise mechanism of action for these proteins is not fully known.

It has been suggested that *Bartonella* GTAs are produced by the fittest cells in a given population in response to cytosolic ppGpp levels (Québatte et al., 2017) and that RcGTA production is also influenced by ppGpp via the RNA polymerase omega subunit (Rpo-ω) (Westbye et al., 2017). *R. capsulatus* Rpo-ω is not required for growth but is essential for RcGTA production (Westbye et al., 2017). In other species, Rpo-ω is thought to play several roles, including stabilization of the RNA polymerase (RNAP) holoenzyme and modulation of transcription profiles via recruitment of alternative sigma factors (Gunnelius et al., 2014; Paget, 2015; Ross et al., 2013; Weiss et al., 2017). One study showed that *Escherichia coli* Rpo-ω can facilitate transcriptional activation when covalently linked to DNA binding proteins (Dove

This is an open access article under the CC BY license (http://creativecommons.org/licenses/by/4.0/).

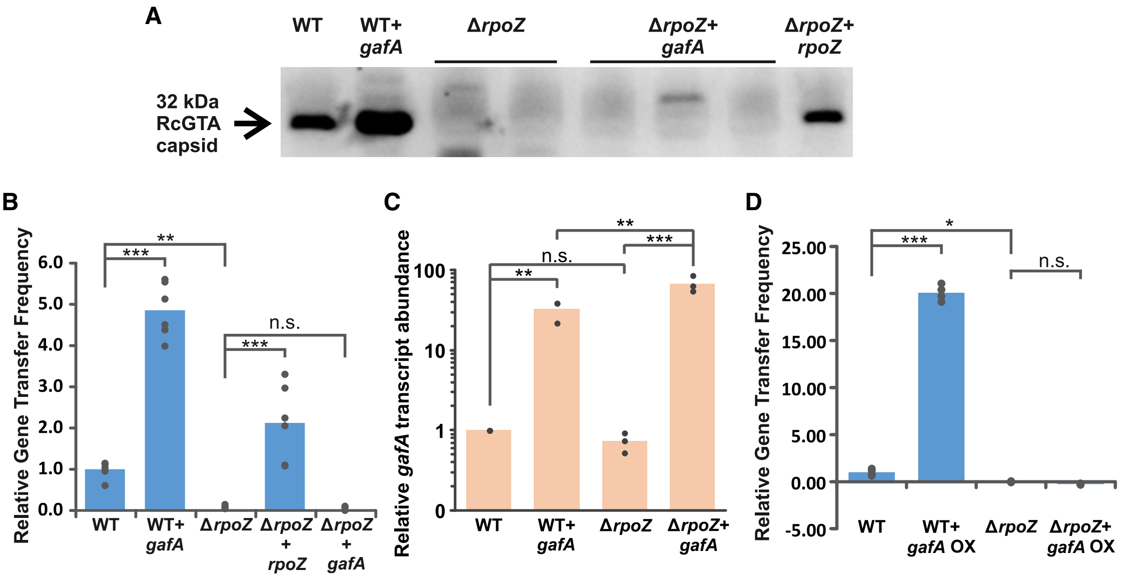

Figure 1. The *rpoZ* gene is essential for RcGTA production

The following strains were used: *R. capsulatus* SB1003 (wild-type [WT]) and a *rpoZ* knockout (KO) derivative (Δ*rpoZ*). Strains were complemented in *trans* with empty pCM66T vector (WT and Δ*rpoZ*), *rpoZ* expressed from its native promoter (+*rpoZ*), *gafA* expressed from its native promoter (+*gafA*), or *gafA* overexpressed from a cumate inducible promoter (+*gafA* OX).

(A) Representative Western blot of *R. capsulatus* concentrated supernatants using an α-RcGTA capsid antibody. See also Data S1.

(B) Bar chart showing the frequency of rifampicin gene transfer by the indicated strains. n = 6.

(C) qPCR data showing *gafA* transcript abundance in the indicated strains relative to the *R. capsulatus* SB1003 (WT) control. Expression levels shown were calculated using the ΔΔCt method (*uvrD* reference gene) and a log2 transformation to give fold differences. n = 3.

(D) Bar chart of the frequency of rifampicin gene transfer by the annotated strains. n = 4.

Statistical significance is indicated on each graph as calculated by one-way ANOVA with the Holm-Sidak method for pairwise multiple comparisons (***$p < 0.001$; **$p < 0.01$; *$p < 0.05$; n.s., $p > 0.05$).

and Hochschild, 1998), but, to our knowledge, no native interaction between Rpo-ω and a transcriptional regulator has ever been demonstrated. Here we examine the relationship between RNAP-ω and the RcGTA activator protein GafA. We explore the protein:protein and protein:DNA binding activities of GafA domains, identify putative *gafA* genes in pathogenic Hyphomicrobiales species, and speculate regarding the overall mechanism of action for the GafA regulator.

RESULTS

Rpo-ω is required for activation of GTA production by GafA

GafA is the only known direct activator of GTA expression in *R. capsulatus* (Fogg, 2019). Rpo-ω, encoded by the *rpoZ* gene, is also required for RcGTA production (Westbye et al., 2017), but the relationship between the two has not been established. Introduction of the plasmid pCMF180, containing *gafA* together with its native promoter, into wild-type *R. capsulatus* SB1003 leads to an increase in RcGTA production, presumably because of increased copy number (Figures 1A and 1B); but deletion of *rpoZ* completely eliminates GTA production (Figures 1A and 1B), both of which corroborate previous findings (Fogg, 2019; Westbye et al., 2017). The pCMF180 plasmid was introduced into SB1003 Δ*rpoZ* to test whether moderate *gafA* overexpression can overcome loss of the RcGTA production phenotype, but no GTA gene transfer was detected (Figure 1B). Western blots of concentrated supernatants using an α-RcGTA capsid antibody also failed to detect any capsid protein accumulation in the supernatant of the SB1003 Δ*rpoZ* + *gafA* strains (Figure 1A). Because *gafA* was expressed from its own promoter, it is possible that Rpo-ω acts to regulate expression of *gafA* and, consequently, the GTA genes indirectly. To confirm that expression of *gafA* was not affected in any way by the loss of *rpoZ*, RNA was extracted for transcript quantification by qPCR. The transcription of *gafA* in SB1003 Δ*rpoZ* was equivalent to the wild-type, and *gafA* transcript abundance was actually higher for SB1003 Δ*rpoZ* + pCMF180 compared with the *rpoZ* replete background (Figure 1C). Finally, a construct was created containing *gafA* expressed from a non-native cumate inducible promoter: pCMF254. Overexpression of *gafA* in SB1003 led to an ~20-fold increase in GTA production, but overexpression in SB1003 Δ*rpoZ* produced no detectable GTA production and was indistinguishable from the empty plasmid control (Figure 1D). These data indicate that Rpo-ω is not required for expression of *gafA* but instead regulates RcGTA production downstream.

Rpo-ω directly interacts with GafA

In multiple species, Rpo-ω is thought to influence RNAP sigma factor preference and, consequently, global gene expression (Gunnelius et al., 2014; Kurkela et al., 2021; Mathew and Chatterji, 2006; Yamamoto et al., 2018). We hypothesized that GafA

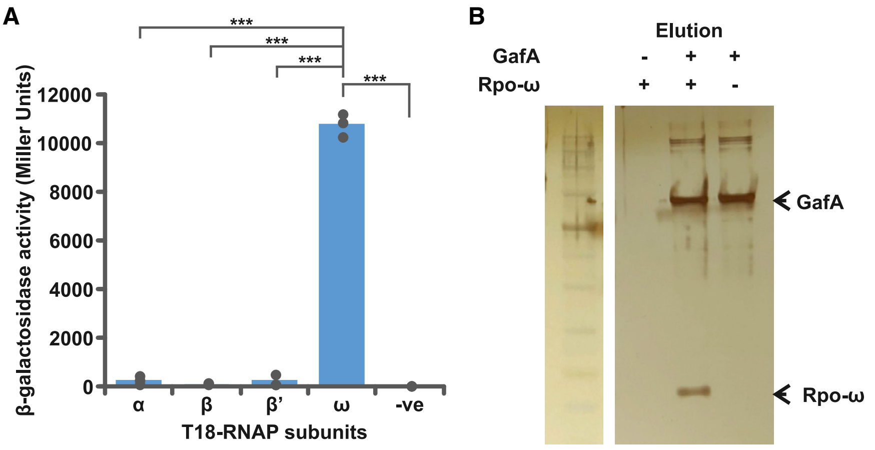

Figure 2. GafA directly interacts with Rpo-ω

(A) Quantification of bacterial 2-hybrid interactions between T25-GafA versus T18-Rpo-α, T18-Rpo-β, T18-Rpo-β', and T18-Rpo-ω. The negative control is T25-gafA versus pUT18 empty vector (−ve). n = 3. Statistical significance is indicated on the graph as calculated by one-way ANOVA with the Holm-Sidak method for pairwise multiple comparisons (***$p < 0.001$).

(B) Silver-stained SDS PAGE gel of a pull-down assay using MBP-GafA as bait and H6-Rpo-ω as prey. Amylose magnetic beads that should only bind to MBP-tagged proteins were used to capture the proteins. The presence or absence of each protein in the assay is indicated by − or + symbols above the gel. The Abcam broad-range protein marker is included for reference.

See also Data S1.

acts by binding to Rpo-ω to alter promoter preferences of the RNAP holoenzyme, and, hence, deletion of *rpoZ* abolishes the influence of GafA. pUT18 bacterial 2-hybrid plasmids were created with each of the *R. capsulatus* RNAP subunits (α, β, β', and ω), and tested for binding to T25-GafA. In this assay, a successful interaction between two proteins brings together the T18 and T25 domains of adenylate cyclase and ultimately leads to production of β-galactosidase, which can be measured using colorimetric substrates such as X-gal or O-nitrophenol (Karimova et al., 1998). The α, β, and β' subunits gave no detectable signal for interaction with GafA, but Rpo-ω produced a strong positive signal in a β-galactosidase assay (Figure 2A). To confirm this result, Maltose Binding Protein-GafA fusion protein (MBP-GafA) (Fogg, 2019) was bound to amylose magnetic beads and used as bait for capture of purified H6-Rpo-ω. Mock bait beads were prepared simultaneously by identical treatment but with GafA protein omitted. Addition of the Rpo-ω protein to mock beads produced no detectable binding, whereas Rpo-ω was detected in the eluate from the GafA pre-bound beads (Figure 2B). These data confirm that GafA interacts directly with Rpo-ω, which is then likely to lead to changes in RNAP promoter selection and specific expression of RcGTA genes.

GafA homologs are present throughout the Rhodobacterales and Hyphomicrobiales

The GafA protein shares little primary sequence similarity with any well-characterized proteins but does possess localized similarity with DnaA and sigma factor DNA-binding domains at the N- and C-terminal regions of the protein (Fogg, 2019). We performed a BLASTp sequence similarity search using the *R. capsulatus* GafA protein as a query, which revealed hits to genes annotated as DUF6456 domain or helix-turn-helix domain-containing proteins from widespread Rhodobacterales species (Table S1A). This agrees with a previous finding showing that GafA homologs were present in all 21 complete Rhodobacterales genomes that were available at that time (Hynes et al., 2016). A recent study by Kogay et al. (2019) proposed that 60% of the 730 available Hyphomicrobiales (formerly Rhizobiales) genome sequences contained putative RcGTA genes, but this study only focused on genes within the core structural gene cluster and so did not include *gafA*. We performed additional PSI-BLAST and BLASTp sequence similarity searches with an *R. capsulatus* GafA protein query but limited the results to the Hyphomicrobiales. Matches were produced with a wide variety of species, but sequence similarity was localized to the C-terminal portion of the protein (Figure S1; Tables S1B and S1C), with the closest sequence similarity found in the final ∼18 kDa. Notably, the majority of Hyphomicrobiales homologs were ∼22–32 kDa compared with the 42-kDa *R. capsulatus* GafA, but in most cases, the "*gafA*" gene was preceded by a small gene predicted to encode a DnaA-like DNA-binding protein (Figure S2). Local synteny was also conserved in the Hyphomicrobiales genomes with a downstream gene predicted to encode a cysteine desulfuration enzyme (*sufE*) and an upstream transcriptional regulator annotated as *mucR* or as a helix-turn-helix containing gene (Figure S2). Occasional exceptions appear to be full-length Rhodobacterales-type *gafA* genes with associated Rhodobacterales synteny or Hyphomicrobiales synteny but without a DnaA-like gene (Figure S2C; Table S1D). Further BLASTp searches with taxonomic limits set for more distantly related Hyphomicrobiales pathogen species (*Agrobacterium* and Brucellaceae) produced similar results in terms of local synteny and sequence identity (Tables S1E and S1F), suggesting that these genes and gene organization are common throughout the order.

The GafA central region is important for protein:protein interactions

We predicted the GafA structure from the primary protein sequence using the AlphaFold program (Jumper et al., 2021). All five AlphaFold models placed the two putative DNA-binding domains in equivalent positions and orientations, linked by a central domain of unknown function (Figures 3Aand3B). Informed by the structural model and the alignments to Hyphomicrobiales genes (Figure S1), three bacterial 2-hybrid constructs were produced using truncated *gafA* gene fragments that encode residues 1–226 (N^{1-226}), 87–382 (Cx^{87-382}), and 221–382 ($C^{221-382}$) (Figure 3A). The three constructs were tested for an interaction with Rpo-ω, and GafA-N^{1-226} and GafA-Cx^{87-382} produced a positive signal but GafA-$C^{221-382}$ did not (Figure 3C). To confirm this result, purified MBP-GafA-Cx^{87-382} protein was bound to amylose magnetic beads and used as bait for capture

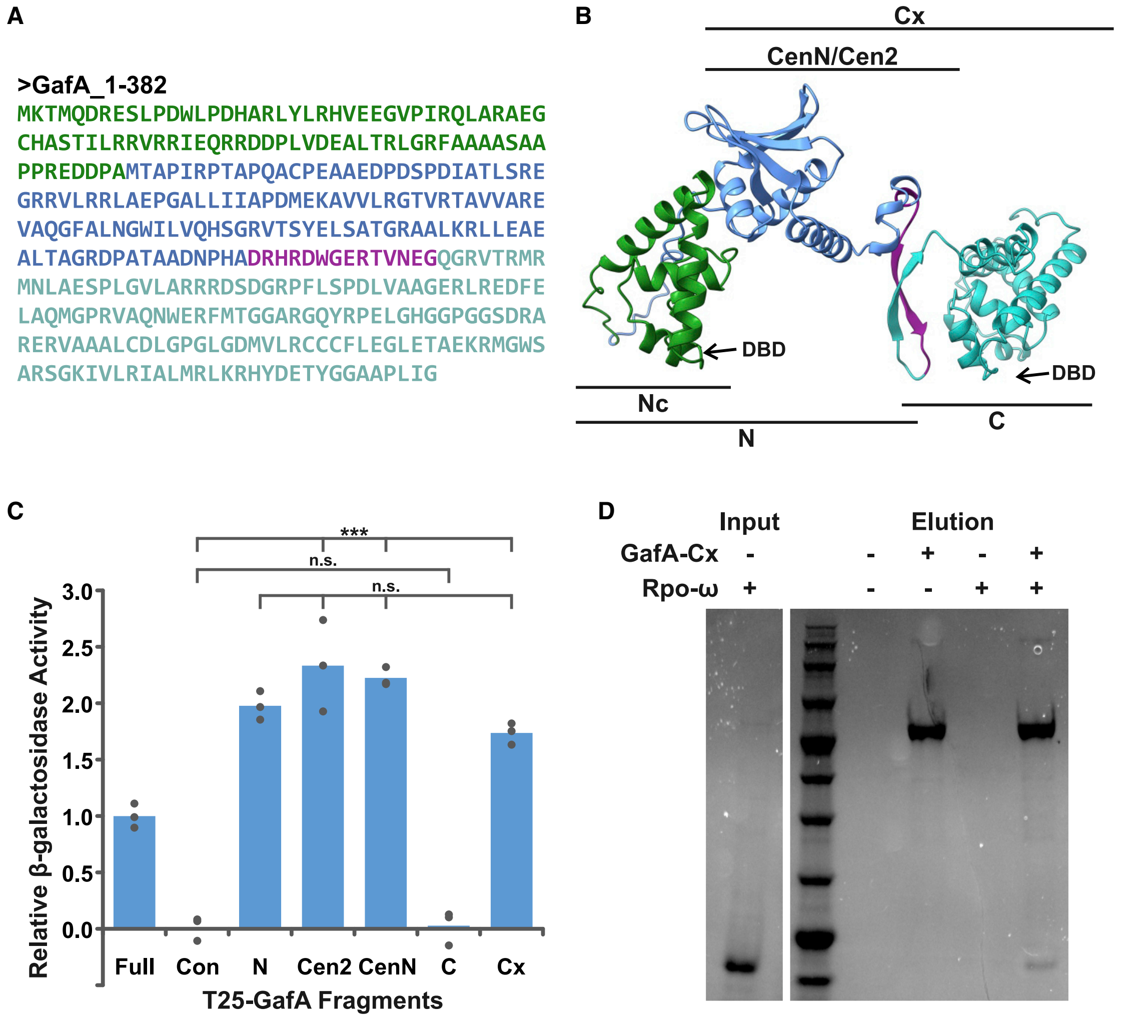

Figure 3. The domain structure of the *R. capsulatus* GafA

(A) Amino acid sequence of GafA, color coded to highlight the different regions used for subsequent characterization. Green, N-terminal concise (Nc, residues 1–86), green and blue, N-terminal (N, residues 1–226), turquoise, C-terminal (C, residues 221–382), blue-turquoise, C-terminal extended (Cx, residues 87–382), blue, central region 2 (Cen2, residues 87–212), blue and purple, central region N (CenN, residues 87–226).

(B) AlphaFold structure prediction for GafA; regions used for subsequent characterization are color coded as in (A) and annotated above and below the image. The two predicted DNA-binding domains (DBDs) are annotated with arrows.

(C) Quantification of bacterial 2-hybrid interactions between T18-Rpo-ω and various T25-GafA constructs (defined above) by β-galactosidase assay. n = 3. Statistical significance is indicated on the graph as calculated by one-way ANOVA with the Holm-Sidak method for pairwise multiple comparisons (***$p < 0.001$; n.s., $p > 0.05$).

(D) InstantBlue-stained SDS-PAGE gel of a pull-down assay using MBP-GafA-Cx as bait and H6-Rpo-ω as prey. Amylose magnetic beads were used to capture the proteins. Presence or absence of each protein in the assay is indicated by – or + symbols above the gel. The Abcam broad-range protein marker and a lane showing the Rpo-ω protein input are included for reference.

See also Figures S1–S4; Tables S1 and S2; and Data S1.

of H6-Rpo-ω in solution. Binding of Rpo-ω to the immobilized MBP-GafA-Cx$^{87–382}$ protein was detected (Figure 3D). The GafA-N$^{1–226}$ and GafA-Cx$^{87–382}$ constructs overlap in the central region of the protein, which suggests that this is the location of GafA:Rpo-ω binding. Additional bacterial 2-hybrid constructs were made to isolate the central region of GafA; i.e., amino acids 87–212 (Cen2) and 87–226 (CenN). Both were positive for binding with Rpo-ω (Figure 3C). These data indicate that GafA is comprised of two distal DNA-binding domains and a central protein-binding domain. The AlphaFold model (Figures 3B and S3) predicted that the central region contains a β sheet motif (amino acids ~129–181) that is presented in the opposite direction to the DNA binding motifs, and we hypothesize that this is the interaction interface for Rpo-ω.

No experimental structures are available for Rpo-ω proteins from species that are closely related to *R. capsulatus.* An HHPRED search, using *R. capsulatus* Rpo-ω as a query, identified structural similarity matches across the first ~70 amino

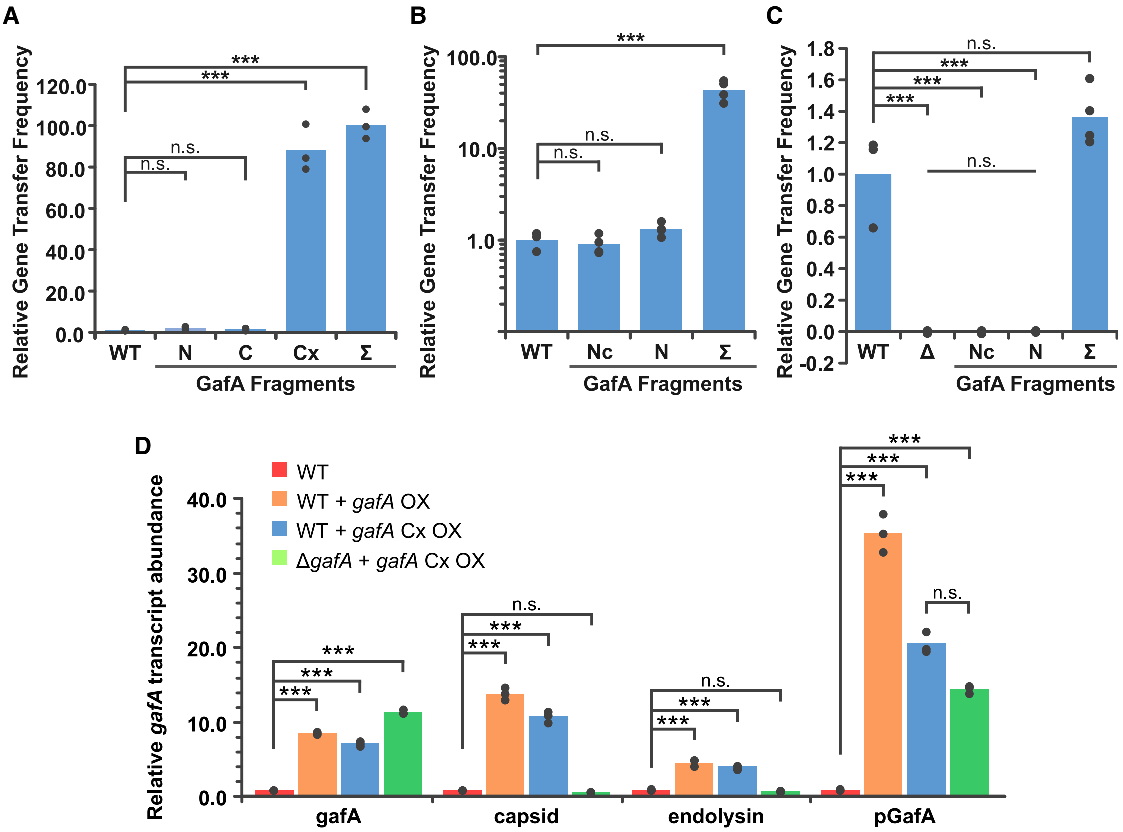

Figure 4. Characterization of GafA domain function

(A–C) Bar charts of the relative frequency of rifampicin gene transfer from (A) *R. capsulatus* SB1003 WT donor strains complemented in *trans* with empty pQF vector (WT), full-length *gafA* (Σ), or truncated regions of *gafA* described in Figure 3 (N, C, and Cx) (n = 3); (B) *R. capsulatus* SB1003 WT donor strains complemented in *trans* with empty pCM66T vector (WT, n = 3) or with the *puf* promoter driving expression of full-length *gafA* (Σ, n = 4), or truncated regions of *gafA* (Nc and N, n = 4); or (C) *R. capsulatus* SB1003 Δ*gafA* donor strains complemented in *trans* with empty pCM66T vector (WT, n = 8) or with the *puf* promoter driving expression of full-length *gafA* (Σ, n = 4) or truncated regions of *gafA* (Nc and N, n = 7).

(D) Transcript abundance of RcGTA genes in *gafA* overexpression strains. The bar chart shows relative changes in transcript abundance measured using qPCR and the ΔΔCt method (n = 3). The *R. capsulatus* strains tested are annotated in the legend: SB1003 containing empty pQF (WT), SB1003 complemented with pCMF254 (WT + *gafA* OX), SB1003 complemented with pCMF264 (WT + *gafA* Cx OX), and SB1003 *gafA* KO complemented with pCMF264. Transcripts of *gafA*, RcGTA capsid (*rcc01687*), RcGTA endolysin (*rcc00555*), and the *gafA* 5′ UTR (*pGafA*) were measured.

Statistical significance is indicated above each graph and was calculated by one-way ANOVA with the Holm-Sidak method for pairwise multiple comparisons (***$p < 0.001$; n.s., $p > 0.05$). See also Figure S5.

acids of the protein (Table S2A). AlphaFold models of *R. capsulatus* Rpo-ω closely matched *E. coli* Rpo-ω but also lacked sufficient confidence at the C terminus (Figures S4A–S4C). The AlphaFold models of Rpo-ω^{1-71} and GafA-CenN were submitted to the LZerD Web Server for protein docking prediction (Christoffer et al., 2021). The results were not conclusive (highest rank sum score = 57), but 6 of the top 10 models predicted that binding occurs with the β sheet (Figure S3D). Further experimental confirmation will be required to definitively pinpoint the binding interface.

GafA N-terminal (NT) is not required for autoregulation but essential for GTA activation

To test whether different domains of GafA play different regulatory roles, three regions shown in Figure 3 (GafA-N^{1-226}, $C^{221-382}$ and Cx^{87-382}) were cloned into the cumate inducible expression vector pQF. The pQF vectors were introduced into the SB1003 wild-type and SB1003 Δ*gafA* strains and tested for various RcGTA production phenotypes. In a RcGTA gene transfer bioassay, GafA-N^{1-226} and GafA-$C^{221-382}$ were unable to induce any RcGTA production in either genetic background (Figures 4A and S5A). Overexpression of GafA-Cx^{87-382} and full-length GafA in wild-type cells stimulated ∼80- to 100-fold greater gene transfer frequencies than the vector-only control (Figure 4A); however, neither was able to complement the *gafA* knockout (Figure S5A). These data were corroborated by visualization of intracellular ∼4-kb RcGTA DNA accumulation by gel electrophoresis (Figure S5B); detection of characteristic bacteriochlorophyll absorbance peaks in cell-free supernatant, indicative of cell lysis (Figures S5C and S5D); and western blots to assess accumulation of the RcGTA capsid in the supernatant (Figure S5E). In all cases, full-length GafA and GafA-Cx^{87-382} induced RcGTA production and lysis in wild-type cells, but no RcGTA production was detected for Δ*gafA* strains complemented with any *gafA* overexpression constructs. The

GafA DnaA-like helix-turn-helix DNA binding motif is very close the N terminus of the protein (amino acids [aa] ~15–55, Figure 3), and so it is possible that extra residues at this end of the protein interfere with DNA binding (Fogg, 2019). Previous work showed that the full-length *gafA* open reading frame (ORF) overexpressed from the *puf* photosynthesis promoter effectively complemented the Δ*gafA* mutant (Fogg, 2019); therefore, we produced comparable *puf*-GafA-Nc$^{1–86}$ and N$^{1–226}$ constructs and introduced them into SB1003 wild-type and SB1003 Δ*gafA* strains. Gene transfer bioassays showed that neither GafA-Nc$^{1–86}$ nor N$^{1–226}$ could complement the *gafA* knockout, and neither could induce RcGTA overexpression in the SB1003 wild-type (Figures 4B and 4C).In *trans* expression of full-length GafA from the *puf* promoter complemented the SB1003 Δ*gafA* strain and increased SB1003 wild-type gene transfer frequencies by 43.5-fold compared with the SB1003 + empty vector control (Figures 4B and 4C).

These data indicate that the presence of a short N-terminal FLAG tag in the pQF vector impaired complementation and that full-length GafA is required to induce RcGTA production. However, the fact that overexpression of a truncated *gafA* completely lacking the N-terminal DNA binding motif still induces high level RcGTA production in the presence of a full-length chromosomal copy of *gafA,* indicates that the GafA-Cx$^{87–382}$ region (Figure 3) can perform at least some of the functions of the full-length protein. We hypothesized that the GafA-Cx$^{87–382}$ portion of the protein can activate the *gafA* promoter independent of the N-terminal DNA-binding domain but that the full protein is required for wider transcriptional activation of other RcGTA genes. To differentiate the effect of full-length GafA and GafA-Cx$^{87–382}$ on RcGTA gene expression in SB1003 wild-type and SB1003 Δ*gafA* cells, the transcript abundance of the RcGTA capsid, endolysin, and *gafA* genes was measured by qPCR (Figure 4D). We used *gafA* primers that bind within the region encoding GafA-Cx, and, therefore, the qPCR measured the total combined transcripts of chromosomal and plasmid-borne *gafA* or *gafA*-Cx genes. As expected, overexpression of *gafA* or *gafA*-Cx$^{87–382}$ in the wild-type or knockout strain produced similar levels of *gafA* transcripts, and, consistent with the phenotypic data, this only led to increased RcGTA capsid and endolysin production in wild-type cells. To quantify the activity of the chromosomal *gafA* promoter, we used primers designed to amplify the 5′ UTR that is present only on the chromosome and is also retained in the Δ*gafA* mutant. Transcription from the native promoter was upregulated more than 10-fold when *gafA* or *gafA*-Cx$^{87–382}$ was overexpressed in wild-type or Δ*gafA* cells (Figure 4D).

Mutation of key residues near the GafA N terminus impairs RcGTA activation

In agreement with previous work (Fogg, 2019), an HHPRED search for structural homologs of GafA identified tentative hits against numerous sigma factors for the predicted N- and C-terminal GafA DNA-binding domains (Tables S2B–S2D; C-terminal DNA-binding domain [DBD], $E > 0.84$; N-terminal DBD, $E \geq 0.0017$). However, the N-terminal DBD also produced hits against three DnaA proteins from diverse species in the PDB, two of which produced E values of 8.5E−07 or greater, as well the DnaA entry from the NCBI conserved domain database (Table S2C). Alignment of the *R. capsulatus* GafA N-terminal region with *E. coli*, *Mycobacterium tuberculosis,* and *Aquifex aeolicus* DnaA proteins showed poor primary sequence conservation overall but patches of increased sequence similarity particularly around the residues predicted to bind in the major groove of DNA (Figure 5A; Blaesing et al., 2000; Fujikawa et al., 2003).

Ten amino acid locations in the GafA protein were chosen based on sequence conservation or predicted involvement in DNA binding (Figures 5A–5C). Each position was changed to alanine in the *gafA* complementation plasmid, pCMF180, by site-directed mutagenesis. The mutated plasmids were introduced into SB1003 Δ*gafA* to assess the relative ability of each to restore RcGTA production. All mutations had a strong effect on protein function with average gene transfer frequencies at approximately 20% or less compared with the unmutated version of *gafA* (Figure 5D). The plasmids were also introduced into a *gafA*-null derivative of the RcGTA overproducer strain *R. capsulatus* DE442. The *gafA* gene is known to be expressed at much higher levels in DE442 than the wild-type SB1003 strain (Fogg, 2019); we hypothesized that a higher dose of some GafA mutants in DE442 might overcome the impaired RcGTA phenotype and reveal which mutations have the greatest effect on function. Most DE442 *gafA* mutants also failed to complement RcGTA production in gene transfer bioassays, with the exception of L34A and L46A (Figure 5E). In our alignment, L34 and L46 correspond to *E. coli* DnaA I425 and L438, neither of which directly bind DNA. In the predicted protein structure, L34 is also located on a separate helix as the major groove DNA-binding residues (Figure 5B). *E. coli* DnaA T435 (equivalent to GafA S43) binds to specific DNA bases, and R442 and K443 (GafA R50 and R51, respectively) interact with the DNA backbone (Figures 5A–5C; Fujikawa et al., 2003). DnaA V437 and Q446 (GafA I45 and Q54, respectively) are not predicted to bind DNA, but they do sit on the same helix as the residues described above; therefore, mutations in this region may affect the general conformation of the binding site. DnaA R399 and S400 (GafA E8 and S9, respectively) bind in the minor groove of DNA (Fujikawa et al., 2003). The AlphaFold model for GafA placed E8 and S9 at a location unlikely to bind DNA (Figure 5B), but this could be due to poor multiple sequence alignment coverage at the protein N terminus (Figure S3).

These data indicate that the truncated GafA-Cx$^{87–382}$ protein can effectively induce expression from the native *gafA* promoter, but full-length GafA is required to induce the various other RcGTA loci. It is likely to be the N-terminal DnaA-like DBD that is essential for activation of the RcGTA promoters.

N- and C-terminal regions of GafA bind DNA

Previous work showed that GafA binds to the RcGTA promoter at a location 75–125 bases upstream of the start codon of TerS (RcGTA *g1/rcc01682*) (Fogg, 2019; Sherlock et al., 2019). The C-terminal 162 aa of GafA was expressed from a T7 expression vector with an N-terminal MBP tag. The protein was purified to homogeneity and used for electrophoretic mobility shift assays (EMSAs) (Figure 6). As predicted, MBP-GafA-C$^{221–382}$ bound the RcGTA promoter at the previously identified location (Figures 6A and 6B), which contains the −10 and −35 promoter elements plus the transcription start site (TSS) (Figure 6A). It is

Cell Press
OPEN ACCESS

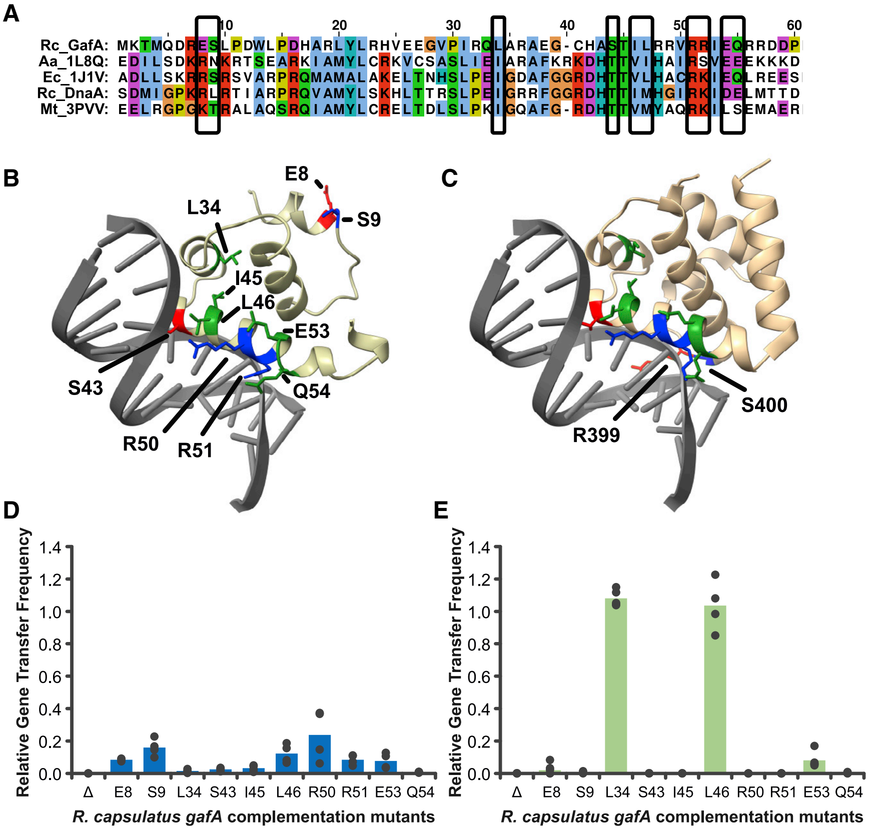

Figure 5. Mutagenesis of the GafA N-terminal DBD

(A) Alignment of *R. capsulatus* GafA (Rc_GafA) residues 1–59 with the DnaA DBDs from *E. coli* (PDB: 1J1V), *M. tuberculosis* (PDB: 3PVV), *A. aeolicus* (PDB: 1L8Q), and *R. capsulatus* (Rc_DnaA). Conserved amino acids are colored using the CLUSTLx scheme, and mutated positions are indicated by black boxes.

(B) AlphaFold structure prediction for the GafA N-terminal DBD. Side chains are shown for the amino acid positions mutated in this study, and each is colored according to predicted interaction with DNA: red, specific base interaction; blue, nonspecific interaction with the DNA backbone; green, no direct interaction.

(C) DBD from *E. coli* DnaA (PDB: 1J1V). Amino acids equivalent to those mutated in GafA are colored using the same scheme as in (B). R399 and S400 are annotated because they sit in the minor groove of DNA, whereas their GafA counterparts (E8/S9) were predicted to have no proximity to the DNA, probably because of limitations of the model at the sequence extremity.

(D and E) Relative gene transfer frequencies for *gafA* gene KO of (D) the WT strain *R. capsulatus* SB1003 (n = 4, except E8, where n = 3) and (E) the RcGTA overproducer strain DE442 (n = 4, except S9, where n = 3). Each strain was complemented in *trans* with empty pCM66T vector (Δ) or the *gafA* gene with single point mutations as indicated on the x axis. Frequencies shown are normalized to complementation of the respective strains (SB1003 or DE442) with unmodified *gafA*. Statistical significance was tested by one-way ANOVA with the Holm-Sidak method for pairwise multiple comparisons. All *gafA* point mutations were statistically different from WT *gafA* ($p < 0.001$) except DE442 complemented with *gafA* L34A or L46A ($p > 0.05$).

also known that *gafA* binds to its own promoter in a 270-base region upstream of the start codon (Fogg, 2019), but the precise location was not identified. To refine the binding site, we used three 50-bp Cy5-labeled dsDNA oligos covering 150 bases upstream of the start codon for an EMSA. GafA-C$^{221–382}$ bound to *gafA* promoter fragment 3, which contains the predicted −35 element (Figures 6D and 6E).

Similar protein expression constructs were also made for the GafA-Nc$^{1–86}$ and GafA-N$^{1–226}$ regions with N-terminal His and MBP tags, but, as expected, no binding was detected for any DNA targets tested, consistent with data (Figure 4) showing that N-terminal modifications probably impair activity of the protein by interfering with DNA binding. To resolve this, the affinity purification tag was removed from the MBP-GafA-Nc$^{1–86}$ protein by digestion with 3c protease, and EMSAs were performed with the tag-free protein. GafA-Nc$^{1–86}$ produced DNA mobility shifts consistent with non-specific binding to most templates (Figure 6). Of the five RcGTA promoter fragments tested, four were bound by GafA-Nc$^{1–86}$ with similar affinities (pGTA 1–4), but only two shifts remained in the presence of an unlabeled non-specific dsDNA competitor (Figure 6C). The two promoter fragments that produced specific binding were located on either side of the GafA-C$^{221–382}$ binding site (Figure 6A), which suggests that GafA could bind as a dimer. Analytic gel filtration confirmed that GafA is dimeric in solution, and dimerization is retained for the truncated GafA-Cx$^{87–382}$ and GafA-C$^{221–382}$ proteins (Figure S6). Of the three *gafA* promoter fragments tested, two were bound by GafA-Nc$^{1–86}$ (pGafA 1 and 2), but neither was specific (Figure 6F), consistent with the observation that the GafA N-terminal 86 amino acids are not required for stimulation of the *gafA* promoter.

DISCUSSION

Production of GTAs is indirectly controlled by various global regulators in response to environmental stimuli, and the disparate signals are integrated via a single transcription factor, GafA. GafA shares little sequence or structural similarity with proteins of known function, but short regions in the N and C termini of the protein have tentative structural similarity to DnaA and sigma factor proteins, respectively (Tables S2B and S2C). These regions of similarity are tightly centered around predicted DBDs. The central portion of GafA, between these putative DBDs, is of unknown function. Here we sought to refine the mechanism of action for GafA and to assign functions to the various domains. We identified a direct interaction between GafA and Rpo-ω.

The interaction between GafA and Rpo-ω

Bacterial DNA-dependent RNAP is responsible for production of all RNA within a given cell. RNAP is a multi-protein holoenzyme comprised of two identical α subunits, catalytic β and β'

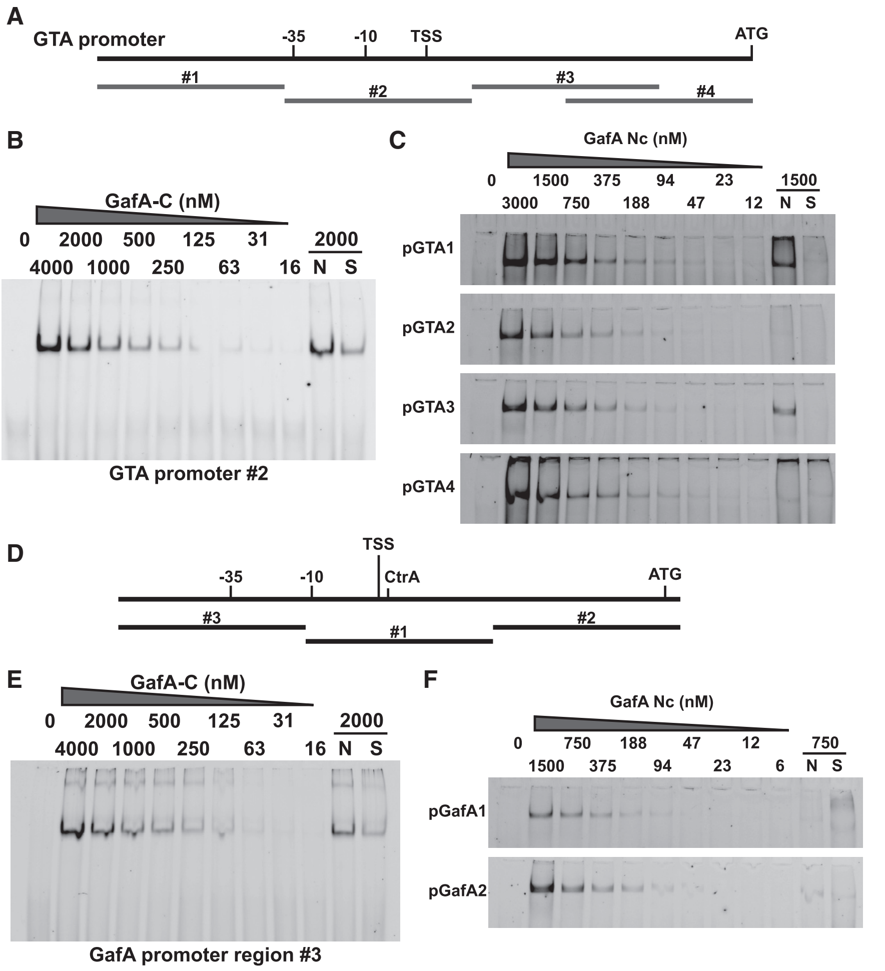

Figure 6. Binding of GafA domains to the RcGTA and *gafA* promoters

(A) Schematic of the RcGTA promoter region with the start codon (ATG), transcription start site (TSS), and −10/−35 promoter elements annotated. The locations of DNA fragments used for EMSA band shifts are shown and labeled 1–4.

(B) Representative EMSA of GafA-C$^{221–382}$ binding to RcGTA promoter fragment 2.

(C) Representative EMSA of GafA- Nc$^{1–86}$ binding specifically to RcGTA promoter fragments 1 and 3 and non-specifically to 2 and 4. Protein concentration is labeled above the image. N, excess of non-specific competitor DNA added; S, excess of specific competitor DNA added.

(D) Schematic of the *gafA* promoter region with the start codon (ATG), TSS, CtrA binding site (CtrA), and −10/−35 promoter elements annotated. The locations of DNA fragments used for EMSA band shifts are shown and labeled 1–3.

(E) Representative EMSA of GafA-C$^{221–382}$ binding to *gafA* promoter fragment 3.

(F) Representative EMSA of GafA- Nc$^{1–86}$ binding non-specifically to *gafA* promoter fragments 1 and 2. Protein concentration is labeled above the image.

See also Figures S6 and S7.

subunits, and an ω subunit encoded by the *rpoZ* gene. The Rpo-ω subunit has been studied in a wide variety of species (Kurkela et al., 2021), where it is thought to stabilize the overall RNAP holoenzyme via direct interactions with the β and β' subunits (Glyde et al., 2018; Lin et al., 2019; Vassylyev et al., 2002). *R. capsulatus* Rpo-ω shares less than 50% sequence identity with its *E. coli* counterpart, but the MAR (Methionine-Alanine-Arginine) ppGpp binding motif and all five conserved residues known to be important for RNAP stabilization are present in both proteins (Kurkela et al., 2021). With the exception of *M. tuberculosis* (Mao et al., 2018), deletion of the *rpoZ* gene is not lethal but results in various growth defects or alternative phenotypes (Kurkela et al., 2021). Westbye et al., 2017 showed that the growth rate of *R. capsulatus* Δ*rpoZ* is slower than that of the wild-type and that RcGTA production is abrogated; the latter was confirmed here (Figure 1).

Evidence from multiple species indicates that deletion of Rpo-ω decreases transcription of some housekeeping genes and influences global transcription profiles by promoting RNAP preference for alternative sigma factors (Paget, 2015; Shimada et al., 2014; Weiss et al., 2017; Yamamoto et al., 2018). The role of Rpo-ω in sigma factor selection has largely been inferred from transcriptome data showing expression profiles characteristic of certain sigma factors in wild-type versus Rpo-ω deletion strains or by the efficiency of sigma factor incorporation into RNAP *in vivo*/*in vitro* (Geertz et al., 2011; Gunnelius et al., 2014; Weiss et al., 2017). Although sigma factors bind to promoter DNA at the −10 and −35 sites, binding is not usually possible *in vitro* in the absence of the RNAP core (Feklístov et al., 2014). Data presented here and elsewhere show that purified GafA does bind *in vitro* to RcGTA promoters close to the −10/−35 regions (Figure 6) and that it is the presence of Rpo-ω rather than its absence that leads to expression of GafA-regulated genes (Figure 1; Fogg, 2019; Westbye et al., 2017). Structural data for RNAP complexes from various species show that the Rpo-ω and sigma factor subunits primarily interact with Rpo-β' but are spatially separated in the stable holoenzyme (Geertz et al., 2011; Glyde et al., 2018; Mao et al., 2018; Vassylyev et al., 2002; Weiss et al., 2017). Transcription factors usually bind upstream of the −35 element and interact with RNAP via the α subunit (Browning and Busby, 2004). No binding was detected between GafA and the Rpo-α, Rpo-β, or Rpo-β' subunits in a bacterial 2-hybrid assay (Figure 2).

Possible scenarios are as follows: (1) GafA first binds to RcGTA promoters and recruits RNAP via an interaction with Rpo-ω, or (2) GafA pre-recruits RNAP in solution and enhances its affinity for RcGTA promoters, similar to the mechanism thought to be used by the MarA/SoxS family (Griffith et al., 2002; Martin et al., 2002). Perhaps binding to Rpo-ω mimicks the action of ppGpp (Westbye et al., 2017), or (3) GafA first binds to Rpo-ω, which then mediates subsequent interactions

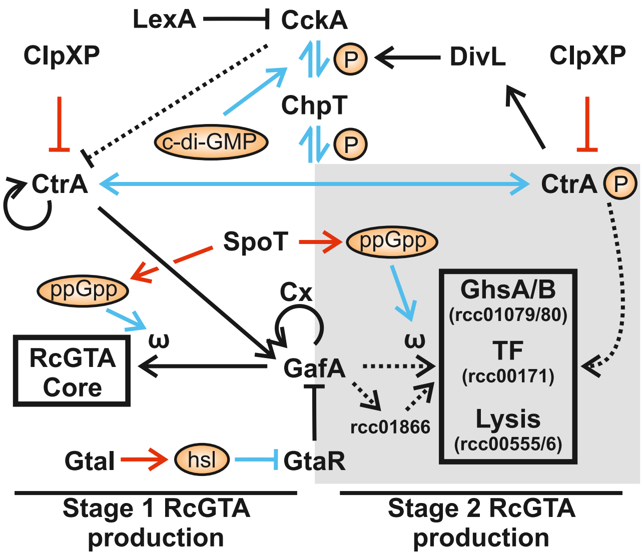

Figure 7. Model of RcGTA regulation

Known contributors to RcGTA regulation are indicated and broadly classified based on whether their major influence is on early production of structural proteins (stage 1) or late-stage maturation and lysis (stage 2). Arrows indicate positive regulation, and flat-headed arrows indicate repression. Black arrows represent transcriptional regulation, blue arrows represent post-translational or ligand:protein regulation, red arrows represent biosynthesis or degradation, and dashed arrows indicate an uncertain mechanism. Arrows representing GafA regulation that requires Rpo-ω are annotated with "ω," and Rpo-ω-independent regulation by the GafA Cx domain is labeled "Cx."

between GafA and the Rpo-α, Rpo-β, or Rpo-β' subunits that are not apparent in one-on-one *in vitro* experiments.

Regulation of the RcGTA operons

We sought to update our previous GafA-centric model for RcGTA (Fogg, 2019) regulation with recent discoveries made here and elsewhere (Figure 7). An important pre-requisite for RcGTA production is high cell density and transition to the stationary phase of growth. The response to cell density is mediated by two contrasting influences: a secreted RTX-domain protein represses expression by an unknown mechanism, whereas a quorum-sensing signal molecule (homoserine lactone [HSL]) promotes RcGTA gene expression (Brimacombe et al., 2013; Ding et al., 2019; Leung et al., 2012; Westbye et al., 2018). Quorum sensing is also important for regulation of the *Dinoroseobacter shibae* GTA (DsGTA), where deactivation of one HSL synthase abolishes any DsGTA gene expression, whereas disruption of another leads to DsGTA overproduction (Koppenhöfer et al., 2019; Tomasch et al., 2018; Wang et al., 2014). A RelA/SpoT homolog responds to amino acid starvation by increasing intra-cellular concentrations of (p)ppGpp, which is likely to interact directly with RNAP via Rpo-ω, or an alternative binding site, to alter promoter preference (Westbye et al., 2017). It is worth noting that *Bartonella* GTA (BaGTA) production appears to occur in response to low ppGpp concentration, leading to the hypothesis that BaGTA production actually occurs in the fittest cells in a population rather than those under the most stress (Québatte and Dehio, 2019; Québatte et al., 2017).

The pleiotropic regulator CtrA is absolutely essential for any detectable RcGTA production, and its phosphorylation state controls the transition from RcGTA assembly and DNA packaging to adornment and lysis (Farrera-Calderon et al., 2021; Lang and Beatty, 2000; Mercer et al., 2010, 2012; Westbye et al., 2013, 2018). Hence, effective production and release of mature RcGTA particles is dependent on an intact phosphorylation cascade from the response regulator CckA to CtrA via ChpT (Farrera-Calderon et al., 2021; Wang et al., 2014; Westbye et al., 2018). High levels of intracellular c-di-GMP stimulate the phosphatase activity of CckA and help to maintain a higher concentration of unphosphorylated CtrA (Farrera-Calderon et al., 2021; Pallegar et al., 2020a, 2020b). In its unphosphorylated state, CtrA is required for transcription of *gafA* (Fogg, 2019). Rpo-ω is not required to activate expression of *gafA*, and only the C-terminal region of GafA is required for autoregulation (Figures 1 and 4), which indicates that different mechanisms regulate transcription of *gafA* and the core RcGTA structural locus. It is likely that GafA works together with CtrA to recruit RNAP to the *gafA* promoter but works alone at the core RcGTA promoter via interaction with Rpo-ω (Figure 7; Fogg, 2019).

Low c-di-GMP levels stimulate CckA kinase activity, leading to CtrA phosphorylation (Farrera-Calderon et al., 2021; Pallegar et al., 2020a, 2020b). CtrA-P also increases expression of the Per-Arnt-Sim (PAS) domain protein DivL, which further enhances CckA kinase activity (Fogg, 2019; Westbye et al., 2018). CtrA-P then acts in concert with GafA to trigger the various late-stage RcGTA genes: head spike (*rcc01079/80*, also known as *ghsA/B*), tail fibers (*rcc00171*), and lysis genes (*rcc00555/6*) (Fogg, 2019). Putative CtrA half-sites were detected in the promoters of all three of these loci and putative GafA binding sites in two of three (Figure S7). The housekeeping protease ClpXP degrades both forms of CtrA and is important for maintenance of a proper equilibrium of phosphorylation states (Westbye et al., 2018). Deletion of ClpX leads to tailless immature RcGTA particles, reminiscent of DNA packaging mutants (Sherlock et al., 2019).

The overall model presented here appears to be mostly complete, with a few notable exceptions. The SoS response regulator LexA is required for RcGTA production, but its precise mechanism is unknown, although it appears to act via CckA (Kuchinski et al., 2016). There is an SOS box in the LexA promoter, and deletion of *lexA* leads to increased expression of *cckA*. This dysregulation presumably unbalances CtrA phosphorylation and/or degradation. LexA, c-di-GMP, CckA, and phosphorylation of CtrA are associated with regulation of DsGTA (Koppenhöfer et al., 2019), but more work is required to fully determine common mechanisms between the species. Another enigmatic protagonist is *rcc01866*, which is located adjacent to the *gafA* gene and is expressed divergently. The Δ*1866* mutant has a phenotype similar to Δ*cckA*; i.e., RcGTA particles are produced but are not fully mature, and no detectable lysis occurs (Hynes et al., 2016). We were unable to predict any putative function for the 1866 protein by primary sequence similarity or structural homology searches.

gafA genes beyond the Rhodobacterales

Through bioinformatics analysis, we identified *gafA* regions with conserved local synteny in the Hyphomicrobiales order

(Figure S2; Tables S1B–S1F). The *gafA* homologs are found in a wide variety of species throughout the order, including several important pathogens, such as those from the *Brucella* (Chain et al., 2011) and *Agrobacterium* genera (Scholz et al., 2008). The *Brucella gafA* genes have also been implicated previously as virulence/fitness factors of unknown function in high-throughput studies (He, 2012; Salmon-Divon et al., 2019). The Hyphomicrobiales *gafA* is split into two separate genes that roughly correspond to the GafA-Nc and GafA-Cx constructs used in this study, supporting the hypothesis that these domains have distinct biological roles.

Our data suggest that GafA acts as an alternative sigma factor or as a transcription factor that is recruited by Rpo-ω via a direct protein:protein interaction (Lane and Darst, 2006; Lin et al., 2019; Li et al., 2019) and that this interaction occurs via the central domain of the protein (Figures 2 and 3). GafA has two mechanisms of action, one Rpo-ω dependent and one Rpo-ω independent, and the GafA N-terminal DBD is essential only for the former. Research is ongoing to determine the precise mechanism of GafA and to establish how widespread this mechanism is in bacteria.

Limitations of the study

The DNA sequence bound by GafA was predicted from the short regions of the GafA and RcGTA promoters identified by EMSA, but a more extensive experimental approach will be required to confirm and refine these predictions; e.g., systematic DNA mutagenesis. Although we demonstrated that GafA interacts with RNAP via the omega subunit to coordinate RcGTA expression, we did not present a definitive mechanism for how RNAP promoter preference is altered. We envisage that this will be resolved via biochemical/structural approaches for the whole RNAP holoenzyme in complex with GafA and DNA.

STAR★METHODS

Detailed methods are provided in the online version of this paper and include the following:

- KEY RESOURCES TABLE
- RESOURCE AVAILABILITY
  - Lead contact
  - Materials availability
  - Data and code availability
- EXPERIMENTAL MODEL AND SUBJECT DETAILS
  - Bacterial strains
- METHOD DETAILS
  - Plasmid construction by cloning and site directed mutagenesis
  - Transformation and conjugation
  - Gene knockouts
  - *Rhodobacter* gene transfer assays
  - Nucleic acid purification
  - Quantitative reverse transcriptase PCR
  - GafA overexpression in *Rhodobacter*
  - Bacterial-two-hybrid (B2H) assays
  - Protein purification
  - Analytical gel filtration
  - Electrophoretic motility shift assays (EMSA)
  - Protein ligand pull down assays
  - Western blotting
  - Sequence similarity analysis
  - Protein structure and function prediction
- QUANTIFICATION AND STATISTICAL ANALYSIS

SUPPLEMENTAL INFORMATION

Supplemental information can be found online at https://doi.org/10.1016/j.celrep.2021.110183.

ACKNOWLEDGMENTS

We thank Prof. Thomas Beatty (University of British Columbia) for critical reading of the manuscript and for his astute suggestion for the GafA-C DNA binding site. We also thank Dr Alexander Westbye (Oslo University Hospital) for critical reading of the manuscript and insightful comments. We acknowledge the important contributions of equipment/technical support from the Genomics and Molecular Interactions labs at the University of York Technology Facility. This research was funded by a Wellcome Trust & Royal Society Sir Henry Dale Fellowship grant (109363/Z/15/A) and a Biotechnology and Biological Sciences Research Council responsive mode grant (BB/V016288/1) (to P.C.M.F.).

AUTHOR CONTRIBUTIONS

Conceptualization, P.C.M.F.; methodology, D.S. and P.C.M.F.; investigation, D.S. and P.C.M.F.; writing, P.C.M.F.; visualization, P.C.M.F.; funding acquisition, P.C.M.F.; resources, P.C.M.F.; supervision, P.C.M.F.

DECLARATION OF INTERESTS

The authors declare no competing interests.

Received: April 5, 2022
Revised: June 6, 2022
Accepted: July 19, 2022
Published: August 9, 2022

REFERENCES

Berglund, E.C., Frank, A.C., Calteau, A., Vinnere Pettersson, O., Granberg, F., Eriksson, A.-S., Näslund, K., Holmberg, M., Lindroos, H., and Andersson, S.G.E. (2009). Run-off replication of host-adaptability genes is associated with gene transfer agents in the genome of mouse-infecting *Bartonella grahamii*. PLoS Genet. *5*, e1000546.

Blaesing, F., Weigel, C., Welzeck, M., and Messer, W. (2000). Analysis of the DNA-binding domain of *Escherichia coli* DnaA protein. Mol. Microbiol. *36*, 557–569.

Brimacombe, C.A., Stevens, A., Jun, D., Mercer, R., Lang, A.S., and Beatty, J.T. (2013). Quorum-sensing regulation of a capsular polysaccharide receptor for the *Rhodobacter capsulatus* gene transfer agent (RcGTA). Mol. Microbiol. *87*, 802–817.

Browning, D.F., and Busby, S.J. (2004). The regulation of bacterial transcription initiation. Nat. Rev. Microbiol. *2*, 57–65.

Chain, P.S.G., Lang, D.M., Comerci, D.J., Malfatti, S.A., Vergez, L.M., Shin, M., Ugalde, R.A., Garcia, E., and Tolmasky, M.E. (2011). Genome of *Ochrobactrum anthropi* ATCC 49188 T, a versatile opportunistic pathogen and symbiont of several eukaryotic hosts. J. Bacteriol. *193*, 4274–4275.

Christoffer, C., Bharadwaj, V., Luu, R., and Kihara, D. (2021). LZerD Protein-Protein docking webserver enhanced with *de novo* structure prediction. Front. Mol. Biosci. *8*, 724947.

CellPress
OPEN ACCESS

Combet, C., Blanchet, C., Geourjon, C., and Deléage, G. (2000). NPS@: network protein sequence analysis. Trends Biochem. Sci. *25*, 147–150.

Ding, H., Grüll, M.P., Mulligan, M.E., Lang, A.S., and Beatty, J.T. (2019). Induction of *Rhodobacter capsulatus* gene transfer agent (RcGTA) gene expression is a bistable stochastic process repressed by an extracellular calcium-binding RTX protein homologue. J. Bacteriol. *201*. e00430–19.

Ding, H., Moksa, M.M., Hirst, M., and Beatty, J.T. (2014). Draft genome sequences of six *Rhodobacter capsulatus* strains, YW1, YW2, B6, Y262, R121, and DE442. Genome Announc. *2*. e00050–14.

Dodd, I.B., and Egan, J.B. (1990). Improved detection of helix-turn-helix DNA-binding motifs in protein sequences. Nucleic Acids Res. *18*, 5019–5026.

Dove, S.L., and Hochschild, A. (1998). Conversion of the omega subunit of *Escherichia coli* RNA polymerase into a transcriptional activator or an activation target. Genes Dev. *12*, 745–754.

Esterman, E.S., Wolf, Y.I., Kogay, R., Koonin, E.V., and Zhaxybayeva, O. (2021). Evolution of DNA packaging in gene transfer agents. Virus Evol. *7*, veab015.

Farrera-Calderon, R.G., Pallegar, P., Westbye, A.B., Wiesmann, C., Lang, A.S., and Beatty, J.T. (2021). The CckA-ChpT-CtrA phosphorelay controlling *Rhodobacter capsulatus* gene transfer agent production is bidirectional and regulated by Cyclic di-GMP. J. Bacteriol. *203*. e00525–20.

Feklístov, A., Sharon, B.D., Darst, S.A., and Gross, C.A. (2014). Bacterial sigma factors: a historical, structural, and genomic perspective. Annu. Rev. Microbiol. *68*, 357–376.

Fogg, M.J., and Wilkinson, A.J. (2008). Higher-throughput approaches to crystallization and crystal structure determination. Biochem. Soc. Trans. *36*, 771–775.

Fogg, P.C.M. (2019). Identification and characterization of a direct activator of a gene transfer agent. Nat. Commun. *10*, 595.

Fogg, P.C.M., Westbye, A.B., and Beatty, J.T. (2012). One for all or all for one: heterogeneous expression and host cell lysis are key to gene transfer agent activity in *Rhodobacter capsulatus*. PLoS One *7*, e43772.

Freese, H.M., Sikorski, J., Bunk, B., Scheuner, C., Meier-Kolthoff, J.P., Spröer, C., Gram, L., and Overmann, J. (2017). Trajectories and drivers of genome evolution in surface-associated marine *Phaeobacter*. Genome Biol. Evol. *9*, 3297–3311.

Fujikawa, N., Kurumizaka, H., Nureki, O., Terada, T., Shirouzu, M., Katayama, T., and Yokoyama, S. (2003). Structural basis of replication origin recognition by the DnaA protein. Nucleic Acids Res. *31*, 2077–2086.

Gabler, F., Nam, S.-Z., Till, S., Mirdita, M., Steinegger, M., Söding, J., Lupas, A.N., and Alva, V. (2020). Protein sequence analysis using the MPI bioinformatics toolkit. Curr. Protoc. Bioinformatics *72*, e108.

Geertz, M., Travers, A., Mehandziska, S., Sobetzko, P., Chandra-Janga, S., Shimamoto, N., and Muskhelishvili, G. (2011). Structural coupling between RNA polymerase composition and DNA supercoiling in coordinating transcription: a global role for the omega subunit? mBio *2*. e00034–11.

Glyde, R., Ye, F., Jovanovic, M., Kotta-Loizou, I., Buck, M., and Zhang, X. (2018). Structures of bacterial RNA polymerase complexes reveal the mechanism of DNA loading and transcription initiation. Mol. Cell *70*, 1111–1120.e3.

Goddard, T.D., Huang, C.C., Meng, E.C., Pettersen, E.F., Couch, G.S., Morris, J.H., and Ferrin, T.E. (2018). UCSF ChimeraX: meeting modern challenges in visualization and analysis. Protein Sci. *27*, 14–25.

Griffith, K.L., Shah, I.M., Myers, T.E., O'Neill, M.C., and Wolf, R.E. (2002). Evidence for "pre-recruitment" as a new mechanism of transcription activation in *Escherichia coli*: the large excess of SoxS binding sites per cell relative to the number of SoxS molecules per cell. Biochem. Biophys. Res. Commun. *291*, 979–986.

Gunnelius, L., Hakkila, K., Kurkela, J., Wada, H., Tyystjärvi, E., and Tyystjärvi, T. (2014). The omega subunit of the RNA polymerase core directs transcription efficiency in cyanobacteria. Nucleic Acids Res. *42*, 4606–4614.

He, Y. (2012). Analyses of *Brucella* pathogenesis, host immunity, and vaccine targets using systems biology and bioinformatics. Front. Cell. Infect. Microbiol. *2*, 2.

Hynes, A.P., Mercer, R.G., Watton, D.E., Buckley, C.B., and Lang, A.S. (2012). DNA packaging bias and differential expression of gene transfer agent genes within a population during production and release of the *Rhodobacter capsulatus* gene transfer agent, RcGTA. Mol. Microbiol. *85*, 314–325.

Hynes, A.P., Shakya, M., Mercer, R.G., Grüll, M.P., Bown, L., Davidson, F., Steffen, E., Matchem, H., Peach, M.E., Berger, T., et al. (2016). Functional and evolutionary characterization of a gene transfer agent's multilocus "genome". Mol. Biol. Evol. *33*, 2530–2543.

Jumper, J., Evans, R., Pritzel, A., Green, T., Figurnov, M., Ronneberger, O., Tunyasuvunakool, K., Bates, R., Žídek, A., Potapenko, A., et al. (2021). Highly accurate protein structure prediction with AlphaFold. Nature *596*, 583–589.

Kaczmarczyk, A., Vorholt, J.A., and Francez-Charlot, A. (2013). Cumate-inducible gene expression system for sphingomonads and other Alphaproteobacteria. Appl. Environ. Microbiol. *79*, 6795–6802.

Karimova, G., Pidoux, J., Ullmann, A., and Ladant, D. (1998). A bacterial two-hybrid system based on a reconstituted signal transduction pathway. Proc. Natl. Acad. Sci. USA *95*, 5752–5756.

Kogay, R., Neely, T.B., Birnbaum, D.P., Hankel, C.R., Shakya, M., and Zhaxybayeva, O. (2019). Machine-learning classification suggests that many alphaproteobacterial prophages may instead be Gene Transfer Agents. Genome Biol. Evol. *11*, 2941–2953.

Kogay, R., Wolf, Y.I., Koonin, E.V., and Zhaxybayeva, O. (2020). Selection for reducing energy cost of protein production drives the GC content and amino acid composition bias in gene transfer agents. mBio *11*. e01206–20.

Koppenhöfer, S., Wang, H., Scharfe, M., Kaever, V., Wagner-Döbler, I., and Tomasch, J. (2019). Integrated transcriptional regulatory network of quorum sensing, replication control, and SOS response in *Dinoroseobacter shibae*. Front. Microbiol. *10*, 803.

Kuchinski, K.S., Brimacombe, C.A., Westbye, A.B., Ding, H., and Beatty, J.T. (2016). The SOS response master regulator LexA regulates the Gene Transfer Agent of *Rhodobacter capsulatus* and represses transcription of the signal transduction protein CckA. J. Bacteriol. *198*, 1137–1148.

Kurkela, J., Fredman, J., Salminen, T.A., and Tyystjärvi, T. (2021). Revealing secrets of the enigmatic omega subunit of bacterial RNA polymerase. Mol. Microbiol. *115*, 1–11.

Lane, W.J., and Darst, S.A. (2006). The structural basis for promoter -35 element recognition by the group IV sigma factors. PLoS Biol. *4*, e269.

Lang, A.S., and Beatty, J.T. (2000). Genetic analysis of a bacterial genetic exchange element: the gene transfer agent of *Rhodobacter capsulatus*. Proc. Natl. Acad. Sci. USA *97*, 859–864.

Lang, A.S., Zhaxybayeva, O., and Beatty, J.T. (2012). Gene transfer agents: phage-like elements of genetic exchange. Nat. Rev. Microbiol. *10*, 472–482.

Leung, M., and Beatty, J. (2013). *Rhodobacter capsulatus* gene transfer agent transduction assay. Bio. Protoc. *3*.

Leung, M.M., Brimacombe, C.A., Spiegelman, G.B., and Beatty, J.T. (2012). The GtaR protein negatively regulates transcription of the *gtaRI* operon and modulates gene transfer agent (RcGTA) expression in *Rhodobacter capsulatus*. Mol. Microbiol. *83*, 759–774.

Li, L., Fang, C., Zhuang, N., Wang, T., and Zhang, Y. (2019). Structural basis for transcription initiation by bacterial ECF σ factors. Nat. Commun. *10*, 1153.

Lin, W., Mandal, S., Degen, D., Cho, M.S., Feng, Y., Das, K., and Ebright, R.H. (2019). Structural basis of ECF-σ-factor-dependent transcription initiation. Nat. Commun. *10*, 710.

Livak, K.J., and Schmittgen, T.D. (2001). Analysis of relative gene expression data using real-time quantitative PCR and the 2(-Delta Delta C(T)) method. Methods *25*, 402–408.

Maniatis, T., Fritsch, E.F., and Sambrook, J. (1982). Molecular Cloning: A Laboratory Manual (Cold Spring Harbor Laboratory Press).

Mao, C., Zhu, Y., Lu, P., Feng, L., Chen, S., and Hu, Y. (2018). Association of ω with the C-Terminal region of the β' Subunit Is essential for assembly of RNA polymerase in *Mycobacterium tuberculosis*. J. Bacteriol. *200*. e00159–18.

Martin, R.G., Gillette, W.K., Martin, N.I., and Rosner, J.L. (2002). Complex formation between activator and RNA polymerase as the basis for transcriptional activation by MarA and SoxS in *Escherichia coli*. Mol. Microbiol. *43*, 355–370.

Mathew, R., and Chatterji, D. (2006). The evolving story of the omega subunit of bacterial RNA polymerase. Trends Microbiol. *14*, 450–455.

McDaniel, L.D., Young, E., Delaney, J., Ruhnau, F., Ritchie, K.B., and Paul, J.H. (2010). High frequency of horizontal gene transfer in the oceans. Science *330*, 50.

Mercer, R.G., Callister, S.J., Lipton, M.S., Pasa-Tolic, L., Strnad, H., Paces, V., Beatty, J.T., and Lang, A.S. (2010). Loss of the response regulator CtrA causes pleiotropic effects on gene expression but does not affect growth phase regulation in *Rhodobacter capsulatus*. J. Bacteriol. *192*, 2701–2710.

Mercer, R.G., Quinlan, M., Rose, A.R., Noll, S., Beatty, J.T., and Lang, A.S. (2012). Regulatory systems controlling motility and gene transfer agent production and release in *Rhodobacter capsulatus*. FEMS Microbiol. Lett. *331*, 53–62.

Motro, Y., La, T., Bellgard, M.I., Dunn, D.S., Phillips, N.D., and Hampson, D.J. (2009). Identification of genes associated with prophage-like gene transfer agents in the pathogenic intestinal spirochaetes *Brachyspira hyodysenteriae, Brachyspira pilosicoli* and *Brachyspira intermedia*. Vet. Microbiol. *134*, 340–345.

Narasimhan, G., Bu, C., Gao, Y., Wang, X., Xu, N., and Mathee, K. (2002). Mining protein sequences for motifs. J. Comput. Biol. *9*, 707–720.

Paget, M.S. (2015). Bacterial sigma factors and anti-sigma factors: structure, function and distribution. Biomolecules *5*, 1245–1265.

Pallegar, P., Peña-Castillo, L., Langille, E., Gomelsky, M., and Lang, A.S. (2020a). Cyclic di-GMP-mediated regulation of gene transfer and motility in *Rhodobacter capsulatus*. J. Bacteriol. *202*. e00554–19.

Pallegar, P., Canuti, M., Langille, E., Peña-Castillo, L., and Lang, A.S. (2020b). A two-component system acquired by horizontal gene transfer modulates gene transfer and motility via cyclic dimeric GMP. J. Mol. Biol. *432*, 4840–4855.

Price, M.N., Wetmore, K.M., Waters, R.J., Callaghan, M., Ray, J., Liu, H., Kuehl, J.V., Melnyk, R.A., Lamson, J.S., Suh, Y., et al. (2018). Mutant phenotypes for thousands of bacterial genes of unknown function. Nature *557*, 503–509.

Québatte, M., and Dehio, C. (2019). *Bartonella* gene transfer agent: evolution, function, and proposed role in host adaptation. Cell Microbiol. *21*, e13068.

Québatte, M., Christen, M., Harms, A., Körner, J., Christen, B., and Dehio, C. (2017). Gene Transfer Agent promotes evolvability within the fittest subpopulation of a bacterial pathogen. Cell Syst. *4*, 611–621.e6.

Ross, W., Vrentas, C.E., Sanchez-Vazquez, P., Gaal, T., and Gourse, R.L. (2013). The magic spot: a ppGpp binding site on *E. coli* RNA polymerase responsible for regulation of transcription initiation. Mol. Cell *50*, 420–429.

Salmon-Divon, M., Zahavi, T., and Kornspan, D. (2019). Transcriptomic analysis of the *Brucella melitensis* Rev.1 vaccine strain in an acidic environment: insights into virulence attenuation. Front. Microbiol. *10*, 250.

Schindelin, J., Arganda-Carreras, I., Frise, E., Kaynig, V., Longair, M., Pietzsch, T., Preibisch, S., Rueden, C., Saalfeld, S., Schmid, B., et al. (2012). Fiji: an open-source platform for biological-image analysis. Nat. Methods *9*, 676–682.

Scholz, H.C., Pfeffer, M., Witte, A., Neubauer, H., Al Dahouk, S., Wernery, U., and Tomaso, H. (2008). Specific detection and differentiation of *Ochrobactrum anthropi, Ochrobactrum intermedium* and *Brucella* spp. by a multi-primer PCR that targets the recA gene. J. Med. Microbiol. *57*, 64–71.

Shakya, M., Soucy, S.M., and Zhaxybayeva, O. (2017). Insights into origin and evolution of α-proteobacterial gene transfer agents. Virus Evol. *3*, vex036.

Sherlock, D., Leong, J.X., and Fogg, P.C.M. (2019). Identification of the first gene transfer agent (GTA) small terminase in *Rhodobacter capsulatus*, its role in GTA production and packaging of DNA. J. Virol. *93*. e01328–19.

Shimada, T., Yamazaki, Y., Tanaka, K., and Ishihama, A. (2014). The whole set of constitutive promoters recognized by RNA polymerase RpoD holoenzyme of *Escherichia coli*. PLoS One *9*, e90447.

Sievers, F., Wilm, A., Dineen, D., Gibson, T.J., Karplus, K., Li, W., Lopez, R., McWilliam, H., Remmert, M., Söding, J., et al. (2011). Fast, scalable generation of high-quality protein multiple sequence alignments using Clustal Omega. Mol. Syst. Biol. *7*, 539.

Solovyev, V., and Salamov, A. (2011). Automatic annotation of microbial genomes and metagenomic sequences. In Metagenomics and its Applications in Agriculture, Biomedicine and Environmental Studies, R.W. Li, ed. (Nova Science Publishers), pp. 61–78.

Tamarit, D., Neuvonen, M.-M., Engel, P., Guy, L., and Andersson, S.G.E. (2018). Origin and evolution of the *Bartonella* gene transfer agent. Mol. Biol. Evol. *35*, 451–464.

Tomasch, J., Wang, H., Hall, A.T.K., Patzelt, D., Preusse, M., Petersen, J., Brinkmann, H., Bunk, B., Bhuju, S., Jarek, M., et al. (2018). Packaging of *Dinoroseobacter shibae* DNA into Gene Transfer Agent particles is not random. Genome Biol. Evol. *10*, 359–369.

Vassylyev, D.G., Sekine, S.I., Laptenko, O., Lee, J., Vassylyeva, M.N., Borukhov, S., and Yokoyama, S. (2002). Crystal structure of a bacterial RNA polymerase holoenzyme at 2.6 A resolution. Nature *417*, 712–719.

Wall, J.D., Weaver, P.F., and Gest, H. (1975). Gene transfer agents, bacteriophages, and bacteriocins of *Rhodopseudomonas capsulata*. Arch. Microbiol. *105*, 217–224.

Wang, H., Ziesche, L., Frank, O., Michael, V., Martin, M., Petersen, J., Schulz, S., Wagner-Döbler, I., and Tomasch, J. (2014). The CtrA phosphorelay integrates differentiation and communication in the marine alphaproteobacterium *Dinoroseobacter shibae*. BMC Genomics *15*, 130.

Waterhouse, A.M., Procter, J.B., Martin, D.M.A., Clamp, M., and Barton, G.J. (2009). Jalview Version 2--a multiple sequence alignment editor and analysis workbench. Bioinformatics *25*, 1189–1191.

Weiss, A., Moore, B.D., Tremblay, M.H.J., Chaput, D., Kremer, A., and Shaw, L.N. (2017). The ω subunit governs RNA polymerase stability and transcriptional specificity in *Staphylococcus aureus*. J. Bacteriol. *199*. e00459–16.

Westbye, A.B., Leung, M.M., Florizone, S.M., Taylor, T.A., Johnson, J.A., Fogg, P.C., and Beatty, J.T. (2013). Phosphate concentration and the putative sensor kinase protein CckA modulate cell lysis and release of the *Rhodobacter capsulatus* gene transfer agent. J. Bacteriol. *195*, 5025–5040.

Westbye, A.B., O'Neill, Z., Schellenberg-Beaver, T., and Beatty, J.T. (2017). The *Rhodobacter capsulatus* gene transfer agent is induced by nutrient depletion and the RNAP omega subunit. Microbiology *163*, 1355–1363.

Westbye, A.B., Kater, L., Wiesmann, C., Ding, H., Yip, C.K., and Beatty, J.T. (2018). The protease ClpXP and the PAS domain protein DivL regulate CtrA and gene transfer agent production in *Rhodobacter capsulatus*. Appl. Environ. Microbiol. *84*. e00275–18.

Wiethaus, J., Wirsing, A., Narberhaus, F., and Masepohl, B. (2006). Overlapping and specialized functions of the molybdenum-dependent regulators MopA and MopB in *Rhodobacter capsulatus*. J. Bacteriol. *188*, 8441–8451.

Wiethaus, J., Schubert, B., Pfänder, Y., Narberhaus, F., and Masepohl, B. (2008). The GntR-like regulator TauR activates expression of taurine utilization genes in *Rhodobacter capsulatus*. J. Bacteriol. *190*, 487–493.

Yamamoto, K., Yamanaka, Y., Shimada, T., Sarkar, P., Yoshida, M., Bhardwaj, N., Watanabe, H., Taira, Y., Chatterji, D., and Ishihama, A. (2018). Altered distribution of RNA polymerase lacking the Omega subunit within the prophages along the *Escherichia coli* K-12 genome. mSystems *3*. e00172–17.

Zimmermann, L., Stephens, A., Nam, S.-Z., Rau, D., Kübler, J., Lozajic, M., Gabler, F., Söding, J., Lupas, A.N., and Alva, V. (2018). A completely reimplemented MPI bioinformatics toolkit with a new HHpred server at its core. J. Mol. Biol. *430*, 2237–2243.

CellPress
OPEN ACCESS

STAR★METHODS

KEY RESOURCES TABLE

REAGENT or RESOURCE	SOURCE	IDENTIFIER
Antibodies		
The anti-RcGTA major capsid protein antibody	Agrisera Ltd	Cat#AS08 365; RRID:AB_1271084
Mouse anti-rabbit IgG-HRP	Santa Cruz Biotechnology	Cat#sc-2357; RRID:AB_628497
Bacterial and virus strains		
Rhodobacter capsulatus: rifampicin resistant strain SB1003	ATCC; www.atcc.org	ATCC BAA-309
Rhodobacter capsulatus: rifampicin sensitive strain B10	(Wall et al., 1975)	B10
Rhodobacter capsulatus: RcGTA overproducer strain DE442	(Ding et al., 2014; Fogg et al., 2012)	DE442
E. coli: S17-1 strain, which contains chromosomally integrated *tra* genes	The Leibniz Institute DSMZ; www.dsmz.de	DSM 9079
E. coli: NEB 10-beta Competent	New England Biolabs	Cat#C3019
E. coli: T7 Express Competent	New England Biolabs	Cat#C2566
Chemicals, peptides, and recombinant proteins		
Phenol:Chloroform:Isoamyl Alcohol 25:24:1 Saturated with 10 mM Tris, pH 8.0	Merck Life Science Limited	Cat#P2069
Chloroform	Merck Life Science Limited	Cat#132950-1L
Proteinase K	Fisher Scientific Ltd	Cat#11456391
RNase A	VWR International	Cat#A2760.0100
BamHI	New England Biolabs	Cat#R3136S
DpnI	New England Biolabs	Cat#R0176L
Q5 DNA polymerase	New England Biolabs	Cat#M0491L
Fast Sybr Green Mastermix	Applied Biosystems	Cat#4385612
Basemuncher Endonuclease	Expedeon Ltd	Cat#ab270049
Imidazole (BioUltra) low UV absorbance	Merck	Cat#56749-250G
Gel filtration molecular weight standard	Biorad	Cat#1511901
InstantBlue Coomassie protein stain	Abcam	Cat#ab119211
Prestained Protein Ladder - Extra broad molecular weight	Abcam	Cat#ab234592
Critical commercial assays		
NEBuilder Cloning Kit	New England Biolabs,	Cat#E5520
NucleoSpin RNA Kit	Macherey-Nagel	Cat#740955
Monarch® PCR & DNA Cleanup Kit	New England Biolabs	Cat#T1030L
Monarch® Plasmid Miniprep Kit	New England Biolabs	Cat#T1010L
LunaScript RT SuperMix Kit	New England Biolabs	Cat#E3010S
Pierce silver stain for mass spectrometry	Thermo Scientific	Cat#24600
SuperSignal west femto maximum sensitivity substrate	Thermo Scientific	Cat#34095
Oligonucleotides		
See Table S4. A complete list of all oligonucleotides used in this study		N/A

(*Continued on next page*)

Continued		
REAGENT or RESOURCE	SOURCE	IDENTIFIER
Recombinant DNA		
See Table S3. A complete list of all the plasmids used in this study		N/A
Software and algorithms		
CorelDraw 2018	Corel Corporation	https://www.coreldraw.com/en/
Sigmaplot version 13	Systat Software Inc.	https://systatsoftware.com/sigmaplot/
Microsoft 365 Suite	Microsoft Corporation	https://www.microsoft.com/en-gb/microsoft-365/microsoft-office
Thermo Fisher Connect	Thermo Fisher Scientific	https://www.thermofisher.com/uk/en/home/digital-science/thermo-fisher-connect.html
FIJI (ImageJ)	Schindelin et al. (2012)	https://imagej.net/software/fiji/
Basic Local Alignment Search Tool	NCBI	https://blast.ncbi.nlm.nih.gov/Blast.cgi
HHpred server	Gabler et al. (2020); Zimmermann et al. (2018)	https://toolkit.tuebingen.mpg.de/tools/hhpred
UCSF ChimeraX version 1.1	Goddard et al. (2018)	https://www.cgl.ucsf.edu/chimerax/
LZerD web server	Christoffer et al. (2021)	https://lzerd.kiharalab.org/about/
NPS@ helix-turn-helix motif prediction	Combet et al. (2000); Dodd and Egan (1990)	https://npsa-prabi.ibcp.fr/cgi-bin/npsa_automat.pl?page=/NPSA/npsa_hth.html
GYM 2.0 helix-turn-helix motif prediction	Narasimhan et al. (2002)	http://users.cis.fiu.edu/~giri/bioinf/GYM2/prog.html
BPROM	Solovyev and Salamov (2011)	http://www.softberry.com/berry.phtml?topic=bprom&group=programs&subgroup=gfindb
Clustal-ω	Sievers et al. (2011)	https://www.ebi.ac.uk/Tools/msa/clustalo/
Jalview version: 2.11.2.2	Waterhouse et al. (2009)	https://www.jalview.org/
Alphafold	Jumper et al. (2021)	https://colab.research.google.com/github/sokrypton/ColabFold/blob/main/beta/AlphaFold2_advanced.ipynb#scrollTo=bQe3KeyTcv0n

RESOURCE AVAILABILITY

Lead contact

Further information and requests for resources and reagents should be directed to and will be fulfilled by the lead contact, Paul Fogg (paul.fogg@york.ac.uk).

Materials availability

All unique reagents or materials generated in this study will be made available on request by the lead contact, but we may require a completed materials transfer agreement if there is potential for commercial application.

Data and code availability

- All data reported in this paper will be shared by the lead contact upon request
- This paper does not report original code
- Any additional information required to reanalyze the data reported in this paper is available from the lead contact upon request.

EXPERIMENTAL MODEL AND SUBJECT DETAILS

Bacterial strains

Three *Rhodobacter capsulatus* strains were used in this study: rifampicin sensitive wild-type strain B10 (Wall et al., 1975), a rifampicin resistant derivative SB1003 (ATCC BAA-309) and an RcGTA overproducer strain DE442 (Ding et al., 2014; Fogg et al., 2012). All *R. capsulatus* cultures were grown at 30°C either aerated in the dark or in anoxic sealed tubes under constant illumination. Two growth media were used – YPS complex broth (0.3% w/v yeast extract, 0.3% w/v peptone, 2 mM $MgCl_2$, 2 mM $CaCl_2$) or RCV defined

broth (10 mM potassium phosphate buffer, 0.4% w/v L-malic acid, 0.1% w/v $(NH_4)_2SO_4$, 0.020% w/v $MgSO_4 \cdot 7H_2O$, 0.0075% w/v $CaCl_2 \cdot 2H_2O$, 0.0012% w/v $FeSO_4 \cdot 7H_2O$, 0.0020% w/v Na_2EDTA, 0.0001% w/v thiamine hydrochloride. Plus 1 mL of trace element solution - 0.07% w/v H_3BO_3, 0.040% w/v $MnSO_4 \cdot H_2O$, 0.019% w/v $Na_2MoO_4 \cdot 2H_2O$, 0.006% w/v $ZnSO_4 \cdot 7H_2O$, 0.001% w/v $Cu(NO_3) \cdot 3H_2O$. The pH was adjusted to 6.8 with NaOH). For agar plates, 1.5% w/v agar was added to the above broth recipes. The *E. coli* S17-1 strain (DSM 9079), which contains chromosomally integrated *tra* genes, was used as a donor for all conjugations. NEB 10-beta Competent *E. coli* cells (New England Biolabs) were used for standard cloning and plasmid maintenance; T7 Express Competent *E. coli* cells (New England Biolabs) were used for overexpression of proteins for purification.

METHOD DETAILS

Plasmid construction by cloning and site directed mutagenesis

A full list of all plasmids and oligonucleotides used in the study can be found in Tables S3 and S4. All oligonucleotides were obtained from Integrated DNA Technologies (IDT) and designed with an optimal annealing temperature of 60°C when used with Q5 DNA Polymerase (New England Biolabs). Plasmid DNA was purified using the Monarch Plasmid Miniprep Kit (New England Biolabs). The destination plasmids pCM66T, pKT25, pUT18 and pUT18C were linearized by digestion with BamHI restriction enzyme (New England Biolabs), pETFPP_1 & 2 was linearized by PCR using inverse primers CleF and CleR. Inserts were amplified using primers with 15 bp 5′ overhangs that have complementary sequence to the plasmid DNA with which it was to be recombined. All cloning reactions were carried out with the NEBuilder Cloning Kit (New England Biolabs). Site-directed mutagenesis was achieved by inverse PCR using Q5 DNA polymerase overlapping primers (offset by 8–10 bp) containing the desired mutation in the center of the overlap region. Amplified DNA was cleaned using the Monarch PCR & DNA Cleanup Kit (New England Biolabs), then digested with DpnI restriction endonuclease (New England Biolabs) overnight at 37°C and introduced without further treatment into chemically competent *E. coli* by transformation.

Transformation and conjugation

Plasmids were introduced into *E. coli* by standard heat shock transformation (Maniatis et al., 1982), and into *Rhodobacter* by conjugation. One milliliter aliquots of overnight cultures of the *E. coli* S17-1 donor and *Rhodobacter* recipient strains were centrifuged at 5,000 x g for 1 min, washed with 1 mL YPS broth, centrifuged again and resuspended in 100 μL YPS broth. 10 μL of concentrated donor and recipient cells were mixed and spotted onto YPS agar or spotted individually as negative controls. Plates were incubated o/n at 30°C. Spots were scrapped, suspended in 100 μL YPS broth and plated on YPS + 100 μg/mL rifampicin (counter-selection against *E. coli*) + 10 μg/mL kanamycin or 1 μg/mL tetracycline (plasmid selection). Plates were incubated o/n at 30°C then restreaked onto fresh selective agar to obtain pure single colonies.

Gene knockouts

Knockouts were created by RcGTA transfer. pCM66T plasmid constructs were created with a gentamicin resistance cassette flanked by 500-1000 bp of DNA from either side of the target gene. Assembly was achieved by a one-step, four component NEBuilder (NEB) reaction and transformation into NEB 10-beta cells. Deletion constructs were introduced into the RcGTA hyperproducer strain and a standard GTA bio-assay (see below) was carried out to replace the intact chromosomal gene with the deleted version.

Rhodobacter gene transfer assays

Rhodobacter assays were carried out essentially as described in (Leung and Beatty, 2013). RcGTA donor cultures were grown photosynthetically (anoxic) with illumination in YPS for ~72 h and recipient cultures were grown under chemotrophic conditions in RCV for ~24 h. Cells were cleared from donor cultures by centrifugation and the supernatant filtered through a 0.45 μm syringe filter. Recipient cells were concentrated 3-fold by centrifugation at 5,000 x g and resuspension in 1/3 volume of G-Buffer (10 mM Tris-HCl pH 7.8, 1 mM $MgCl_2$, 1 mM $CaCl_2$, 1 mM NaCl, 0.5 mg/mL BSA). Reactions were carried out in polystyrene culture tubes (Starlab) containing 400 μL G-Buffer, 100 μL recipient cells and 100 μL filter donor supernatant, then incubated at 30°C for 1 h. 900 μL YPS was added to each tube and incubated for a further 3 h. Cells were harvested by centrifugation at 5,000 x g and plated on YPS + 100 μg/mL rifampicin (for standard GTA assays) or 3 μg/mL gentamicin (for gene knock-outs).

Nucleic acid purification

One milliliter samples of relevant bacterial cultures were taken for each nucleic acid purification replicate. Total DNA was purified according to the "Purification of Nucleic Acids by Extraction with Phenol:Chloroform" protocol (Maniatis et al., 1982). Cells were resuspended in 567 μL TE then 30 μL 10% (w/v) SDS and 3 μL 20 μg/mL Proteinase K were added. Cells were incubated at 37°C for 1 h. To each tube, 100 μL of 5 M NaCl was added and thoroughly mixed by inversion. Eighty microlitres of 1% (w/v) CTAB was added, mixed thoroughly by inversion and the cells were incubated at 65°C for 10 min. An equal volume of Phenol:chloroform:isoamyl alcohol (25:24:1, pH 8) was added and mixed vigorously. The tubes were centrifuged at 15,000 x g for 10 min. The upper aqueous layer was removed to a fresh tube and the Phenol:chloroform:isoamyl alcohol treatment was repeated at least two times or until the white interphase was no longer visible. An equal volume of chloroform was added and mixed vigorously. The tubes were centrifuged at 15,000 x g for 2 min. The upper aqueous layer was transferred to a fresh tube and DNA was precipitated by addition of 0.6 volumes of ice-cold

isopropanol. Precipitation was allowed to proceed at −20°C for 1 h. DNA was harvested by centrifugation at 15,000 x g for 15 min, and the supernatant was discarded. The pellet was washed with 70% ethanol, centrifuged at 15,000 x g for 15 min and the supernatant was discarded. The pellet was allowed to air dry for ~15 min then resuspended in TE buffer. Total RNA was purified using the NucleoSpin RNA Kit (Macherey-Nagel) and DNAseI treated on column according to the recommended protocol. RNA was quantified using a Nanodrop spectrophotometer. 1 μg of total RNA was converted to cDNA using the LunaScript RT SuperMix Kit (NEB).

Quantitative reverse transcriptase PCR

One in fifty dilutions of the cDNA template were prepared and 1 μL used per reaction. Reactions contained Fast Sybr Green Mastermix (Applied Biosystems), cDNA and primers (500 nM). Standard conditions were used with an annealing temperature of 60°C. All primer efficiencies were calculated as between 90 and 110%. Relative gene expression was determined using the ΔΔCt method (Livak and Schmittgen, 2001). For each sample, variance was calculated for three independent biological replicates, which were each the mean of three technical replicates. QuantStudio 3 Real-Time PCR System was used for all experiments (Applied Biosystems).

GafA overexpression in *Rhodobacter*

Gene overexpression in *Rhodobacter* was achieved by a transcriptional fusion of the genes of interest to a cumate inducible promoter in the plasmid pQF (Kaczmarczyk et al., 2013) or to the *R. capsulatus puf* photosynthesis promoter in pCM66T (Fogg, 2019; Fogg et al., 2012). For overexpression experiments using the *puf* promoter, donor cultures were first grown chemotrophically in the presence of oxygen to stationary phase then diluted 1:1 in fresh media and switched to anoxic photosynthetic growth for 6 h. pQF was a gift from Julia Vorholt (Addgene plasmid #48095). Overexpression was induced by addition of cumate to late log growth phase cultures at a final concentration of 50 μM.

Bacterial-two-hybrid (B2H) assays

The procedure and the resources were as described in (Karimova et al., 1998). Plasmids encoding T18 (pUT18C and derivatives) and the compatible plasmids encoding T25 (pKT25 and derivatives) were introduced pairwise into competent BTH101 by co-transformation. Selection was using LB agar containing 50 μg/mL kanamycin, 100 μg/mL ampicillin, 1 mM IPTG and 80 μg/mL X-Gal, and plates were incubated at 30°C for 48 h to allow blue color to develop. Colonies obtained from the B2H plate assay were used to inoculate 5 mL of LB supplemented with 50 μg/mL kanamycin, 100 μg/mL ampicillin and 1 mM IPTG in a 96-well plate. Plates were incubated for 16 h at 30°C with agitation. Absorbance (OD_{600}) readings of culture density were taken. Meanwhile, 80 μL aliquots of permeabilization solution (100 mM Na_2HPO_4, 20 mM KCl, 2 mM $MgSO_4$, 0.5 mg/mL lysozyme) were mixed with 20 μL of each bacterial culture, then incubated at room temperature for 30 min. Six hundred microliters of substrate solution (60 mM Na_2HPO_4, 40 mM NaH_2PO_4, 1 mg/mL ONPG) was added and the mixture was incubated at room temperature. Once sufficient color had developed, stop solution (1 M sodium carbonate) was added and the precise time noted. Cell debris was removed by centrifugation and absorbance (OD_{420}) readings were taken. Miller units were calculated according to the formula MU = 1,000(Abs420/(Abs600*0.02 mL*time in min)).

Protein purification

For His6-tagged proteins, 500 mL cultures of *E. coli* containing the relevant expression plasmid were induced at mid-exponential growth phase with 0.2 mM IPTG overnight at 20°C (Fogg and Wilkinson, 2008). Concentrated cells were lysed in 20 mL binding buffer (0.5 M NaCl, 75 mM Tris; pH 7.75) plus 0.2 mg mL^{-1} lysozyme and 500 U Basemuncher Endonuclease (Expedeon Ltd.) for 30 min on ice and then sonicated. Cleared supernatant was applied to a 5 mL HisTrap FF crude column (Cytiva) and the bound, his-tagged protein was eluted with 125 mM imidazole. Eluted protein was desalted on a HiPrep 26/10 desalting column (Cytiva) and then further separated by size exclusion chromatography on a HiLoad 16/60 Superdex 200 preparative grade gel filtration column. All chromatography steps were carried out on an AKTA Prime instrument (Cytiva). Purified proteins were concentrated in a Spin-X UF Centrifugal Concentrator (Corning) and quantified by the nanodrop extinction co-efficient method (Thermo Scientific). Samples were stored at −80°C in binding buffer plus 50% glycerol. MBP-tagged proteins were purified as above except MBP binding buffer was used (200 mM NaCl, 20 mM Tris, 1 mM EDTA; pH 7.4), the lysate was applied to a 5 mL MBPTrap FF column (Cytiva) and purified protein was eluted with 10 mM maltose in binding buffer.

Analytical gel filtration

Protein multimeric states were estimated using a Superdex 200 increase 10/200 GL column (Cytiva). MBP binding buffer was used for all filtration runs. A protein molecular weight standard (1.3–670 kDa, Bio-Rad Laboratories) was run through the column at 0.75 mL/min and the peaks produced were used to construct a standard curve ($R^2 = 1$, predicted error for 17-670 kDa is <2%). Samples of each protein were sequentially run on the column and molecular weights were estimated from the elution volume and the equation derived from the standard curve.

Electrophoretic motility shift assays (EMSA)

For all 50 bp binding substrates, 50 base Cy5 5′-labelled oligos (IDT) were annealed to unlabelled complimentary oligos (IDT). Both oligos were mixed to a final concentration of 40 μM in annealing buffer (1 M Potassium Acetate, 300 mM HEPES; pH 7.5) and heated to 98°C for 5 min then allowed to cool to room temperature. Ten microliter EMSA mixtures contained 80 nM annealed Cy5-dsDNA,

standard binding buffer (25 mM HEPES, 50 mM K-glutamate, 50 mM $MgSO_4$, 1 mM dithiothreitol, 0.1 mM EDTA, 0.05% Triton X-100; pH 8.0) (Wiethaus et al., 2008), 1 μg poly dI:dC, 4% glycerol and the specified concentrations of purified protein (Wiethaus et al., 2006). 500-fold excess of competitor DNA was added to control mixtures – specific competitor was unlabelled but otherwise identical to the binding substrate and the non-specific competitor was an unlabelled 50 bp annealed oligo matching an arbitrary location elsewhere in the *R. capsulatus* genome. All assays were incubated for 30 min at room temperature then immediately loaded onto a 7% Acrylamide gel (1 x TBE) without loading dye. Gels were run at 80 V for 90 min at room temperature in 1 x TBE. Fluorescence was imaged using a Typhoon Biomolecular Imager (Amersham) and analyzed using ImageQuant (Amersham) and FIJI (Schindelin et al., 2012) software.

Protein ligand pull down assays

One hundred microliters of 2 mg/mL MBP-tagged protein in binding buffer (200 mM NaCl, 20 mM Tris, 1 mM EDTA; pH 7.4) was incubated with 100 μL of amylose magnetic beads (New England Biolabs) at 4°C for 1 h on a rolling platform. Mock beads were created by an identical method but using 100 ul of binding buffer without protein. Beads were washed 5 times with 500 μL of wash buffer (binding buffer + 0.05% Tween 20) and resuspended in a final volume of 100 μL. For pull downs, 25 μL of prepared beads were harvested in a magnetic stand and the supernatant was replaced with either 100 μL of binding buffer alone or binding buffer containing 2 mg/ml H6-RpoZ. The beads were incubated for 2 h at 4°C on a rolling platform, then washed five times with wash buffer. To elute proteins, 50 μL of elution buffer was added (binding buffer + 10 mM maltose). LDS buffer (Abcam) was added to the eluate and heated to 90°C for 10 min. Twenty microliters of each sample were run on a 4–20% TruPAGE denaturing gradient gel (Merck Life Science Ltd) and the bands were visualized using Pierce silver stain for mass spectrometry (Thermo Scientific) or InstantBlue Coomassie stain (Abcam). Five microliters of extra broad molecular weight prestained protein ladder was used for size estimation (Abcam).

Western blotting

Rhodobacter capsulatus supernatants were concentrated 10-fold using a SpeedVac (Thermo Scientific). Fifteen microliter samples were mixed with 5 μL LDS sample buffer (Abcam). heated to 90°C for 10 min and then run on 4–20% TruPAGE denaturing gradient gel (Merck Life Science Ltd). Proteins were transferred to a PVDF membrane using a Mini-PROTEAN Tetra Cell blotting module (Bio-Rad Laboratories) in 1X transfer buffer (25 mM tris base, 0.2 M glycine, 20% methanol; pH8.5), 100 V for 1 h. The membrane was blocked in 5% (w/v) skimmed milk powder in 1X TBS for 1 h at room temperature. The anti-RcGTA major capsid protein antibody (Agrisera Ltd) was used at 1:1000 dilution in blocking buffer overnight at 4°C, followed by four 10 min washes in TBST. The secondary HRP-antibody conjugate was used at 1:2500 dilution in blocking buffer for 2 h at room temperature, followed by four 10 min washes in TBST. SuperSignal west femto maximum sensitivity substrate (Thermo Scientific) was used to develop the western and the signal was detected using an iBRIGHT chemi-imager (Thermo Scientific).

Sequence similarity analysis

NCBI BLASTp and PSI-BLAST searches for GafA homologues were performed using the default parameters - expect threshold = 0.05, word size = 6 or 3 (respectively), blosum62 similarity matrix, gap costs of existence = 11 and extension = 1. Queries were made against the non-redundant protein sequences database (nr; posted:May 5th 2022). Where indicated, taxonomic constraints were applied to limit results to the Hyphomicrobiales (taxid:356), Brucellaceae (taxid:118882) and Agrobacterium (taxid:357). A tBLASTn search was made using a GafA homologue from Roseibium sp. RKSG952 as a query and using the default parameters - expect threshold = 0.05, word size = 6, blosum62 similarity matrix, gap costs of existence = 11 and extension = 1. The nucleotide collection database was used (nr/nt; May 9th 2022 update). A summary of the full outputs can be found in Table S1.

HHPRED analysis of GafA was carried out using the "pdb_mmcif70_14_Apr" and "NCBI_Conserved_Domains(CD)_v3.18" databases accessed on the eighth May 2022 (Gabler et al., 2020; Zimmermann et al., 2018). Full length GafA protein sequence and two shorter sequences, focused on the two predicted DNA binding domains, were used as queries. The default parameters were used in each case i.e. HHBlits UniRef30 MSA generation method, maximal generation steps = 3 and an E-value threshold of 1e-3. Minimum coverage was 20%, minimum sequence identity was 0%. Secondary structure scoring was done during alignment. A summary of the full outputs can be found in Table S2.

Protein structure and function prediction

Three-dimensional structures for the *R. capsulatus* GafA and Rpo-ω proteins were predicted using the Alphafold co-lab server using the msa_method:jackhammer and all other parameters set to default (Jumper et al., 2021). GafA predictions were made on 30th Sept 2021 and RpoZ predictions were made on 3rd February 2022. Protein structures were visualized using the UCSF ChimeraX version 1.1 (Goddard et al., 2018). Protein:protein interaction predictions were produced using the LZerD protein docking algorithm on the LZerD web server using default parameters (Christoffer et al., 2021). Helix-turn-helix predictions were carried out using NPS@ (Combet et al., 2000; Dodd and Egan, 1990) and Gym2.0 (Narasimhan et al., 2002) using the default settings. Promoter −10/−35 elements were predicted with BPROM (Solovyev and Salamov, 2011). Clustal-ω (Sievers et al., 2011) was used for DNA/protein alignments and Jalview version: 2.11.2.2 (Waterhouse et al., 2009) was used to visualize these alignments; relevant similarity/identity color schemes are indicated in the figure legends.

QUANTIFICATION AND STATISTICAL ANALYSIS

CorelDraw 2018 (Corel Corporation) was used for figure preparation Statistical analysis was carried out using Sigmaplot software version 13 (Systat Software Inc.) and, for each use, the test parameters are indicated in the figure legends and, where appropriate, in the main text. All graphs present the means as a bar chart and the individual data points are overlaid as discrete dots. All N values quoted refer to distinct biological replicates.

Cell Reports, Volume *40*

Supplemental information

The archetypal gene transfer agent RcGTA
is regulated via direct interaction
with the enigmatic RNA polymerase omega subunit

David Sherlock and Paul C.M. Fogg

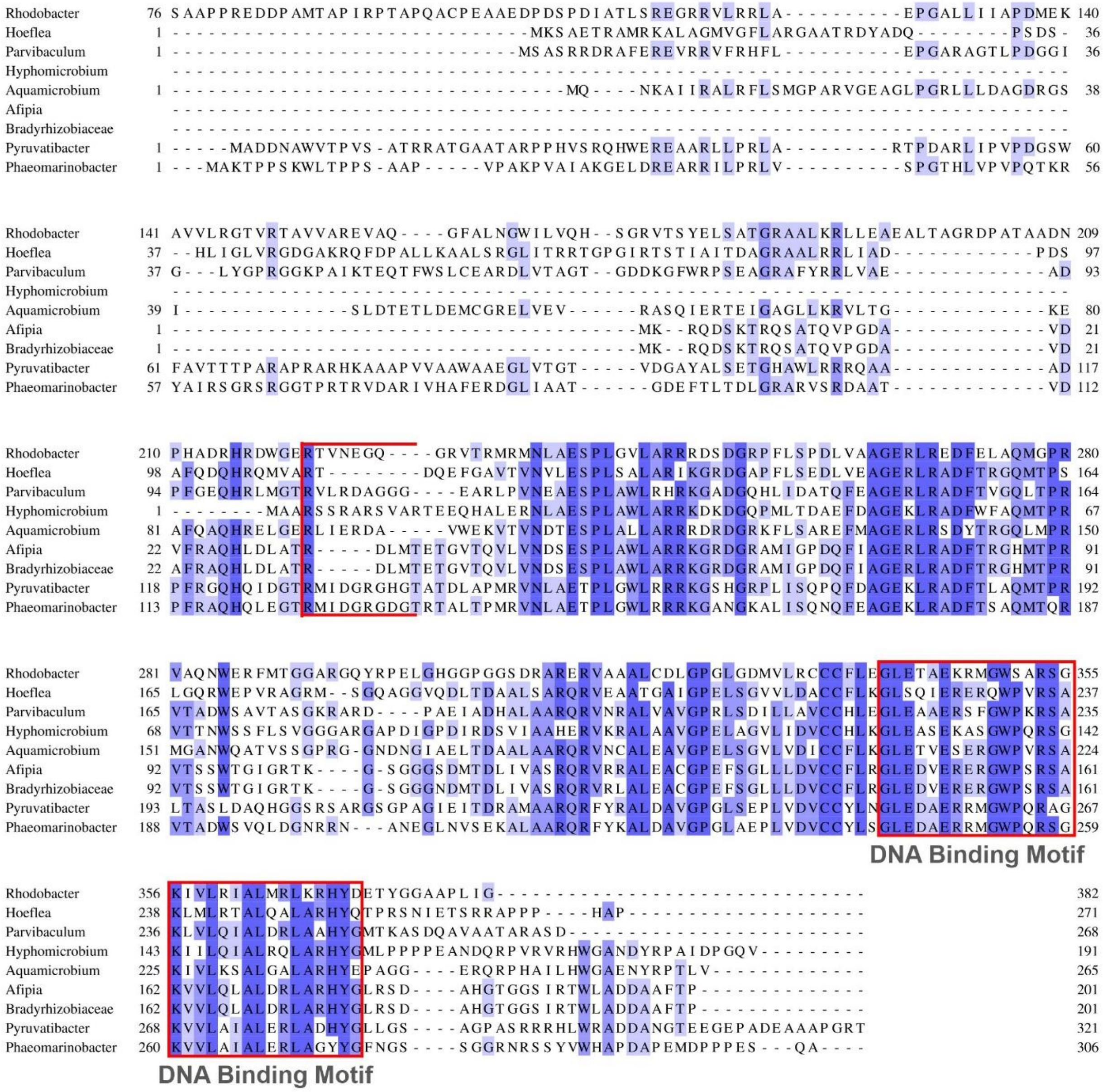

Figure S1. Alignment of the *R. capsulatus* GafA C-terminal extended domain with Hyphomicrobiales counterparts. Related to Figure 3. The top four hits against fully assembled Hyphomicrobiales genomes were chosen from separate BLASTp and PSI-BLAST sequence similarity searches with an *R. capsulatus* GafA query. Conservation is indicated with the Jalview percentage identity colour scheme. The predicted C-terminal DNA binding domain is boxed and annotated to highlight increased sequence conservation. The open box indicated the beginning of the C-terminal concise constructs.

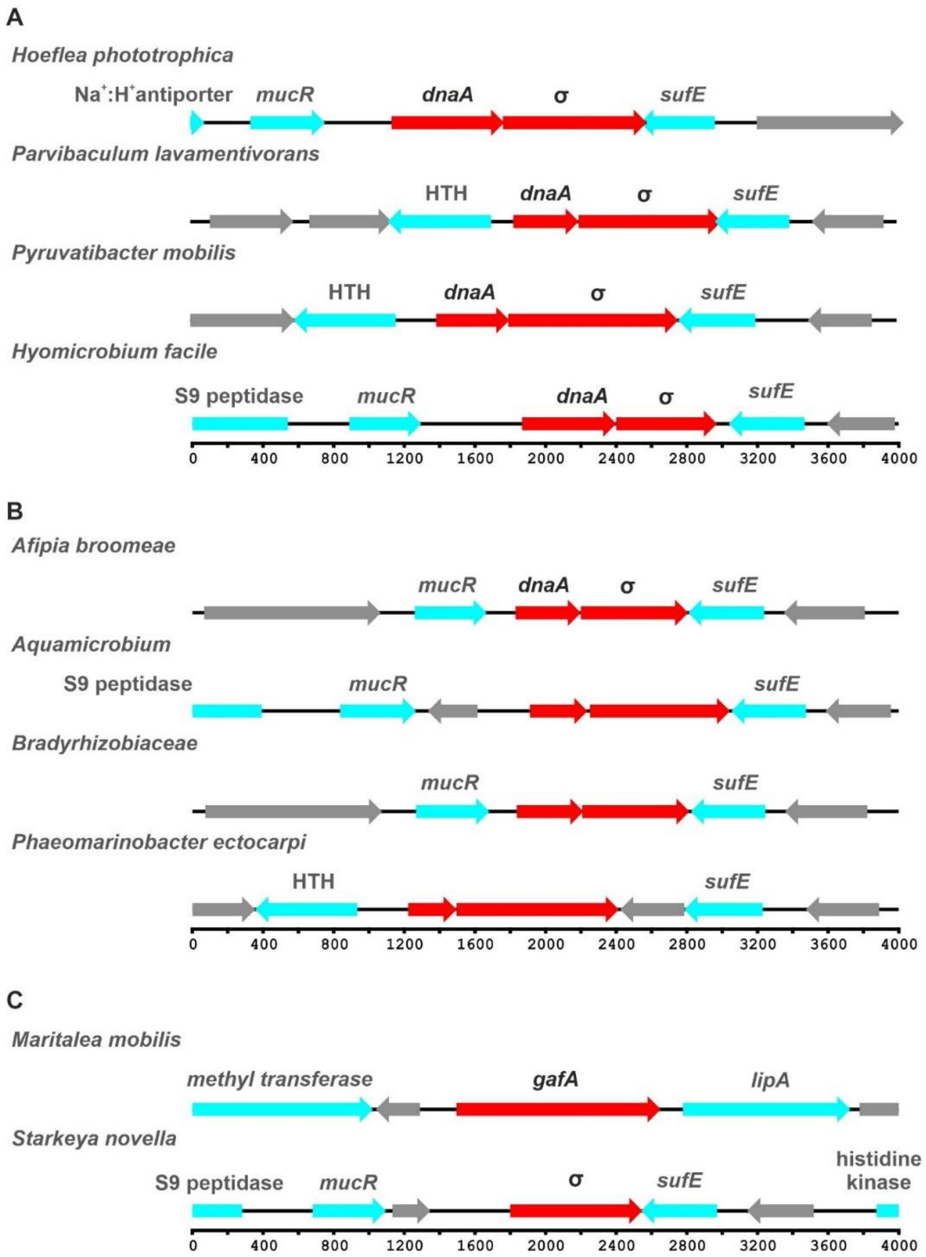

Figure S2. Synteny plots for Hyphomicrobiales GafA homologues. Related to Figure 3. The top four **A.** BLASTp and **B.** PSI-BLAST hits for *R. capsulatus* GafA against fully assembled Hyphomicrobiales genomes. Sequence matches mainly occurred for the GafA C-terminal region only with genes annotated as DUF6456 domain-containing proteins. The matched Hyphomicrobiales genes are annotated here using the HHPRED prediction of a Sigma factor-like domain (σ) and the ORF is coloured red. The upstream dnaA-like ORF is also coloured red. Flanking genes with predicted function are cyan, hypothetical proteins of unknown function are grey. **C.** Two exceptions are shown where either a full-length match was obtained but with Rhodoabcterales-like synteny (*Maritalea*) or the *dnaA* gene was absent with otherwise Hyphomicrobiale-like synteny (*Starkeya*). Scale bars are provided below each panel in bases.

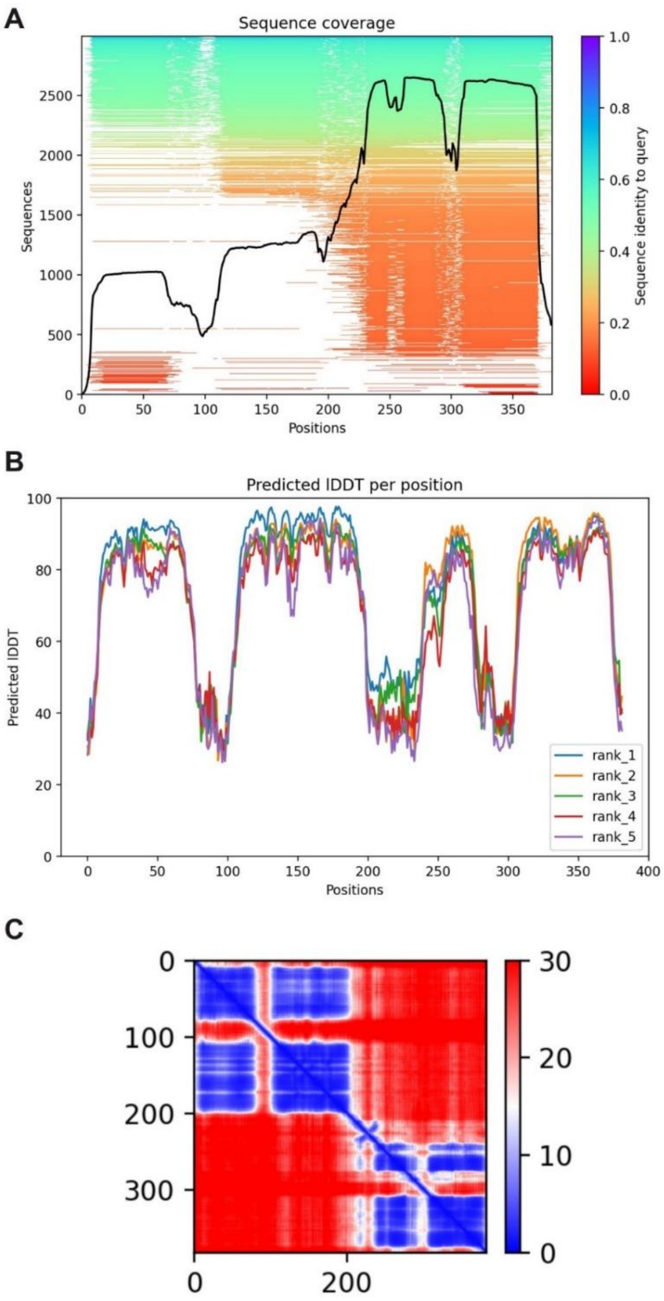

Figure S3. Confidence outputs for *R. capsulatus* GafA structure prediction. Related to Figure 3. A. The jackhmmer method was used on the Alphafold server to align GafA to related proteins and the multiple sequence alignment coverage plot is shown. Aligned sequence coverage is depicted as a line chart and sequence identity is colour coded as shown in the legend. **B.** AlphaFold output plot showing the predicted local Distance Difference Test score (pIDDT) confidence metric. Amino acid positions are shown on the X-axis. **C.** Predicted Aligned Error for each amino acid position labelled on the X and Y-axes. Error is shown on a scale of 0-30, and colour coded as shown in the legend. Clear drop-offs in model confidence can be seen between predicted domains, but each domain is has strong scores typically >80.

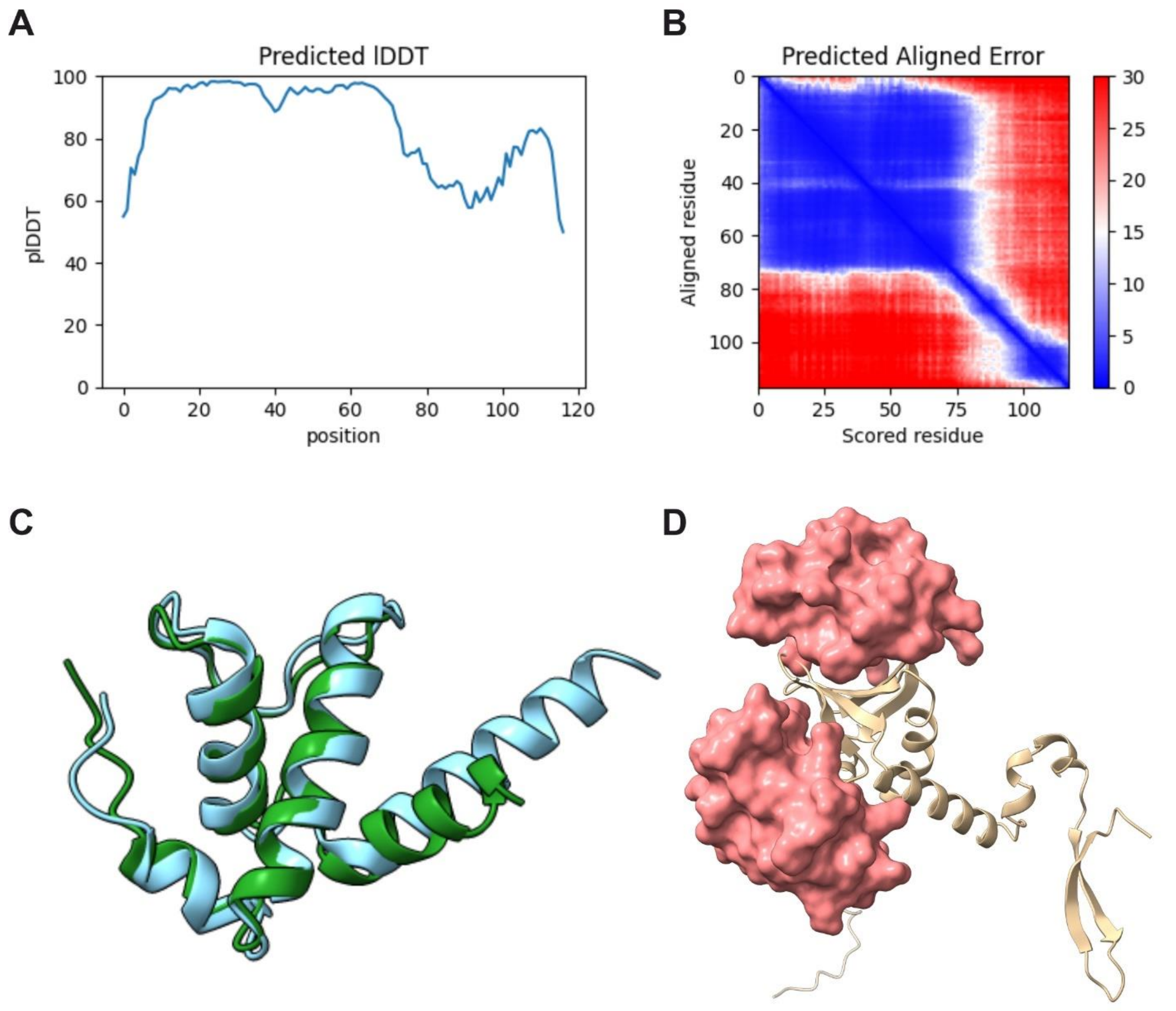

Figure S4. Predicted structure of *R. capsulatus* Rpo-ω protein and its interaction with GafA. Related to Figure 3. A & B. AlphaFold output plots showing the predicted local Distance Difference Test score (pIDDT) confidence metric and Predicted Aligned Error for each amino acid position. A clear drop-off in model confidence, domain packing and broader topology is observed from approximately residue 70 onwards. **C.** AlphaFold predicted *R. capsulatus* Rpo-ω structure trimmed to residues 1-71 (green) and overlaid with *E. coli* Rpo-ω, PDB: 6ALF (pale blue). **D.** LZerD protein docking predictions for GafA-CenN and Rpo-$\omega^{1\text{-}71}$. The two Rpo-ω surface structures shown are representatives of the two centroid clusters that comprise the top ten interaction models. The upper location in contact with the β-sheet was favoured by 6 out of 10 models including the top ranked (rank sum = 47).

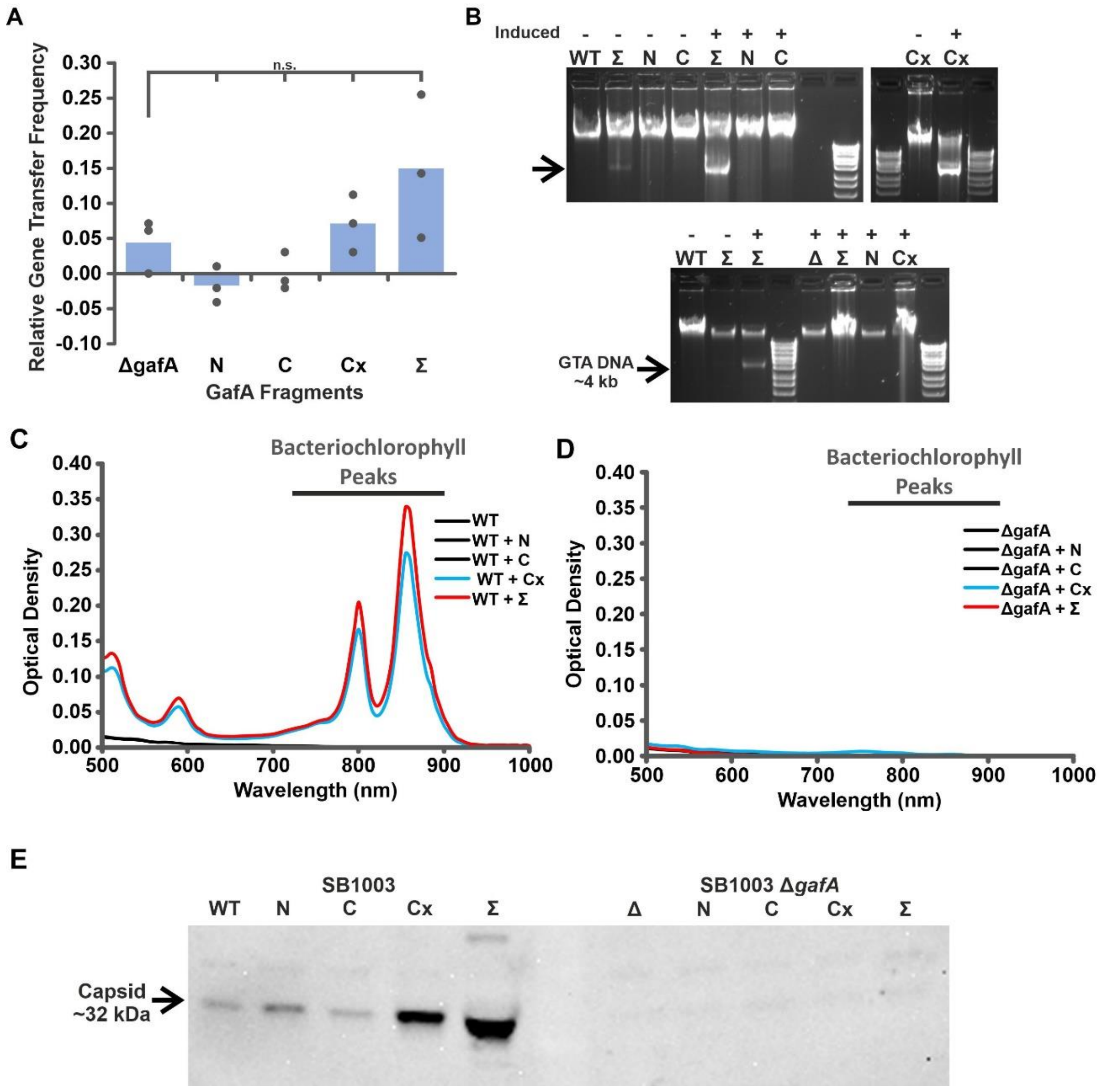

Figure S5. RcGTA production phenotypes after *in trans* expression of GafA full length and truncated proteins. Related to Figure 4. In all panels, SB1003 wild-type and a Δ*gafA* derivative were complemented with empty pQF vector (WT or Δ) or pQF containing truncated *gafA* genes as indicated (*gafA*-N, *gafA*-C, *gafA*-Cx, *gafA*-Σ). **A.** Chart of the frequency of rifampicin gene transfer from *R. capsulatus* SB1003 Δ*gafA* donor strains complemented *in trans* with the indicated pQF vectors, N = 3. **B.** Total intracellular DNA content showing the presence or absence of characteristic 4 kb RcGTA DNA. **C.** Mean absorbance trace of *R. capsulatus* SB1003 supernatant or **D.** SB1003 Δ*gafA* supernatants in the 500-1000 nm wavelength range. Complementation *in trans* the pQF plasmid containing full-length *gafA* is represented by a red line, with *gafA-Cx* is represented by a cyan line and all other constructs (pQF-empty, *gafA*-N and *gafA*-C) are shown in black. N=6 except Δ*gafA* + Cx N=4. Distinctive bacteriochlorophyll peaks indicating cells lysis are annotated. **E.** Representative western blot of concentrated supernatant from the indicated *R. capsulatus* strains using an α-RcGTA capsid antibody. See also Data S1.

A

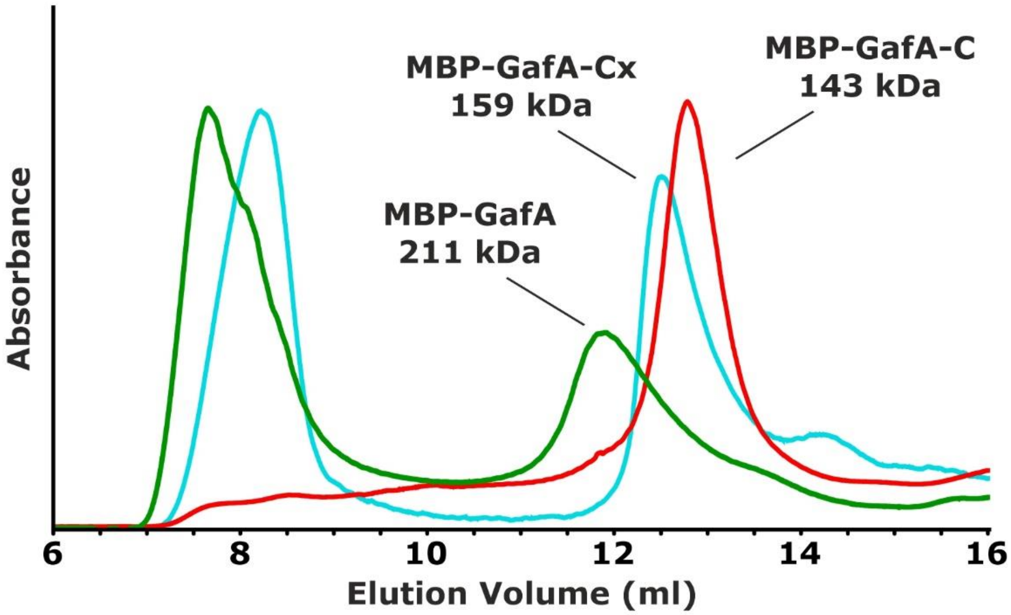

B

Protein	Elution Peak (ml)	Estimated MW (kDa)	Monomer Size (kDa)	Ratio
**MBP-GafA-Cx**	12.54	158,779	75,806	2.1
**MBP-GafA-C**	12.78	142,537	61,119	2.3
**MBP-GafA**	11.91	210,774	85,159	2.5

Figure S6. Analytical gel filtration of GafA proteins. Related to Figure 6. A. Representative traces showing absorbance of GafA (green), GafA-Cx (cyan) and GafA-C (red) at 280 nm versus elution time from the column. Absorbance values are omitted on the Y-axis because the traces are scaled differently to improve comparability. **B.** Summary table of values plotted in part A, the estimated MW of the protein peaks, the calculated MW of each monomer and the ratio of observed MW to that of the monomer.

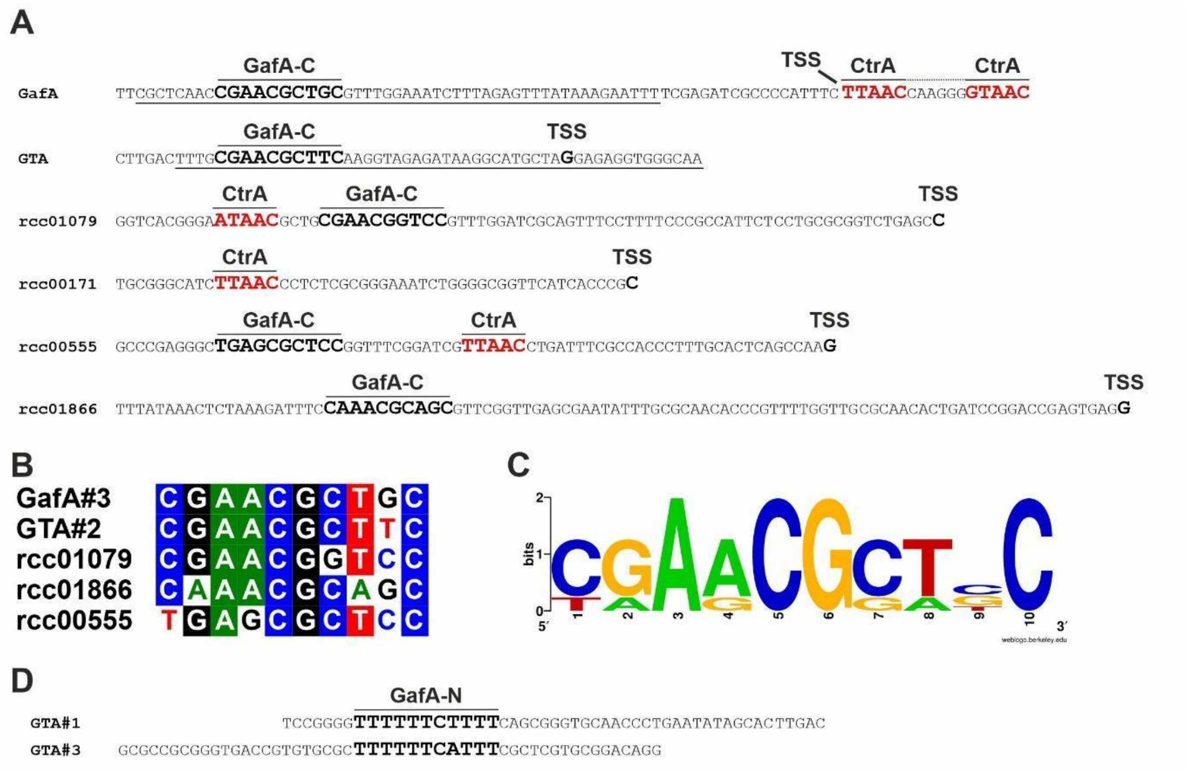

Figure S7. Predicted binding sites for GafA N/C-terminal DNA binding domains. Related to Figure 6. **A.** Schematic of RcGTA related promoters. Transcription start sites (TSS) were estimated based on published RNAseq data. Predicted CtrA binding sites/half-sites are highlighted in bold red and annotated, predicted GafA C-terminal (GafA-C) DNA binding sites are highlighted in bold black and annotated. Underlined sequence indicates the region used for EMSA band shift assays. The five predicted GafA-C binding sites are depicted in **B.** an alignment and **C.** a Logo plot. **D.** The two oligo sequences that were specifically bound by the GafA N-terminal DNA binding domain (GafA-N) are shown with the putative binding site aligned, emboldened and annotated.
